# Clone-specific residue changes at multiple positions are associated with amyloid formation by antibody light chains

**DOI:** 10.3389/fimmu.2025.1622207

**Published:** 2025-08-01

**Authors:** Gareth J. Morgan, Tatiana Prokaeva

**Affiliations:** ^1^ Boston University Amyloidosis Center, Boston University Chobanian & Avedisian School of Medicine, Boston, MA, United States; ^2^ Section of Hematology and Medical Oncology, Department of Medicine, Boston University Chobanian & Avedisian School of Medicine, Boston, MA, United States; ^3^ Department of Pathology and Laboratory Medicine, Boston University Chobanian & Avedisian School of Medicine, Boston, MA, United States

**Keywords:** systemic AL amyloidosis, protein aggregation, amyloidogenesis, immunoglobulin light chains, somatic hypermutation, rare disease, plasma cells

## Abstract

**Introduction:**

Systemic AL amyloidosis is caused by deposition of monoclonal antibody light chains (LC) as insoluble amyloid fibrils in multiple tissues, leading to irreversible and eventually fatal organ damage. Each patient has a unique LC sequence that appears to define its propensity to aggregate. The complexity and diversity of LC sequences has impeded efforts to understand why some LCs aggregate to cause disease while others do not.

**Methods:**

We investigated residue changes, relative to the inferred precursor germline sequences, in monoclonal LCs associated with AL amyloidosis and multiple myeloma (MM), derived from the AL-Base resource. Consensus matrices, calculated using healthy polyclonal repertoire sequences from Observed Antibody Space (OAS), were used to determine the relative frequency of each residue in the monoclonal LC sequences.

**Results:**

A subset of residues observed in AL-associated LCs was uncommon in the healthy repertoire, but these residues were highly diverse and were also observed in MM-associated LCs. We identified multiple positions that more frequently harbor uncommon residues in AL-associated LCs than OAS-derived LCs, including several positions that have previously been identified. However, each individual residue change occurs in only a small fraction of LCs, indicating that many types of residue change can contribute to disease. Furthermore, positions where residue changes occur most frequently were not enriched in amyloidosis-associated residues.

**Discussion:**

These data provide a framework for future investigations into sequence determinants of amyloid propensity, supporting efforts towards earlier recognition and diagnosis of AL amyloidosis.

## 1 Introduction

Aggregation of amyloid fibrils derived from antibody light chain (LC) proteins leads to systemic AL amyloidosis (1). This incurable disease causes progressive organ failure due to displacement of healthy tissue and other toxic mechanisms (2, 3). Amyloid-forming LCs are secreted from monoclonal B cells, most commonly plasma cells that aberrantly proliferate in the bone marrow (2, 3). However, AL amyloidosis is rare and most individuals with an expanded population of such clonal plasma cells, known as monoclonal gammopathy or plasma cell dyscrasia (PCD), do not experience clinically significant amyloid deposition (4). In multiple myeloma (MM), a plasma cell cancer, circulating LC levels are high but amyloid deposition is relatively rare and generally does not cause symptoms (5). Transcriptomic analysis of plasma cells has not identified consistent differences between AL amyloidosis and MM, although both diseases involve similar genomic structural variants (6). Instead, the sequence and properties of the secreted monoclonal LC are hypothesized to determine risk of amyloidosis (7–10). LCs that can aggregate as amyloid fibrils are said to be “amyloidogenic”, but the sequence and structural determinants of amyloidogenicity are not well understood (11). Predicting which monoclonal LCs carry a risk of aggregation would allow earlier diagnosis of AL amyloidosis, potentially leading to improved patient outcomes. Therefore, it is important to understand how amyloidogenic LCs differ from those of the healthy immune repertoire and MM clones.

The major challenge to such a prediction is the diversity of LC sequences. LCs are components of antibodies that have undergone recombination and somatic hypermutation in response to antigen (8). A typical LC accumulates 5–15 amino acid residue changes, relative to the germline-encoded variable, joining and constant precursor genes (*IGV_L_*, *IGJ_L_* and *IGC_L_*, respectively) from which it was derived (11). These mutations are primarily created by activation-induced cytidine deaminase, leading to a characteristic pattern of mutations that differs between precursor genes (12, 13). Healthy humans have an estimated repertoire of 10^6–^10^7^ unique LC sequences, several orders of magnitude less than the heavy chain (HC) repertoire, but far more complex than most other proteins (14).

All LCs must fold to their native state in order to be secreted from plasma cells (15). LCs form two structural domains with similar immunoglobulin folds, variable (V_L_) and constant (C_L_), corresponding to the *IGV_L_-IGJ_L_* and *IGC_L_* gene sequences, respectively. Sequence variation is concentrated in the three complementarity determining regions (CDRs 1–3) of the V_L_ domain, which form the antigen binding site. However, residue changes in framework regions (FRs 1–4), which account for the remainder of the V_L_ domain sequence, also contribute to LC diversity. Peptides from the V_L_ domain, in non-native conformations, form the core of most AL amyloid fibrils (9, 16–23). Residue changes in the C_L_-domain can also influence amyloidogenicity (23–26), but these are uncommon and less well characterized because most studies have focused on V_L_ domains. C_L_ domain sequences are often not determined or reported.

Several hundred monoclonal LC sequences associated with AL amyloidosis and other PCDs, primarily MM, have been determined (27–34). These sequences are collected in AL-Base, a public repository run by Boston University Amyloidosis Center (11, 35). A subset of precursor variable genes (*IGKV* and *IGLV*, encoding κ and λ LCs, respectively) is more common in AL amyloidosis than in MM or the polyclonal immune repertoire (11, 27, 28, 36). Notably, around 75% of AL clones secrete a λ LC, compared to 40% of MM clones (11). Among healthy individuals, approximately one third of circulating antibodies has a λ LC (37). One precursor gene, *IGLV6-57*, accounts for 18% of AL LCs, making it the most frequently used precursor, versus 1% of MM clones and 2% of the healthy repertoire (11). In contrast, *IGKV1-33*, *IGLV1-44*, *IGLV2-14* and *IGLV3-1* collectively account for 44% of AL clones but are also relatively common in MM (27% of clones) and the polyclonal repertoire (17% of sequences) (11). Therefore, specific residue changes, relative to the germline sequences of these genes, appear to predispose LCs towards or away from amyloid formation.

Two approaches to investigating the roles of LC sequence in AL amyloidosis are to compare multiple sequences (11, 36, 38–40) and to investigate the effects of specific residue changes on a single sequence (41–44). In both cases, a key challenge is selecting the most appropriate control sequences. Most investigators have used germline or MM LCs as supposedly “non-amyloidogenic” controls. Germline LCs represent an unmutated, wild-type-like reference, whereas MM LCs are, like AL LCs, produced from aberrant plasma cells after selection for antigen binding. However, both types of LC can be induced to aggregate *in vitro*, although this typically occurs less readily than for AL LCs (43–46). Neither control is ideal due to the number of residue differences between any two LCs, since the effect of each position depends on the rest of the sequence. A further problem is that the identity of the germline gene can only be unambiguously determined from the genome sequence, which is not generally available. Sequences derived from mRNA or peptides must be aligned to the germline gene with the highest homology. This is complicated by paralogs and multiple alleles of most germline genes (8, 47, 48). Therefore, identifying the effect of individual residue changes—and thereby predicting the properties of a new sequence—is not possible without extensive biochemical characterization (41–44).

Individual residue changes may destabilize the LC native state, enhance formation of amyloid fibrils or both (41–44). However, it is difficult to extrapolate beyond the specific LC that was studied. Only a few residue changes are known to contribute to amyloidogenicity in more than one LC. Examples include the R25G polymorphism in the *IGLV6-57* gene, which alters the stability of the native state (46), and N-glycosylation of asparagine 86 in LCs derived from *IGKV1*-family genes (38, 49), although the mechanisms by which N-glycosylation promote amyloid formation are unknown (50). More recent attempts to identify amyloidogenic sequences have used machine learning algorithms to distinguish amyloid from non-amyloid LCs (51–54). However, these algorithms performed less well on a test cohort of several hundred sequences that we compiled following the recent revision of AL-Base (11).

Here, we analyzed the recently extended AL-Base data (11), as well as a larger set of polyclonal sequences from the Observed Antibody Space (OAS) resource (55, 56). We evaluated the frequency and positions of individual residue changes that are observed in AL amyloidosis, relative to MM and the polyclonal repertoire. By creating consensus matrices for each precursor gene from thousands of OAS sequences, we estimated the likelihood of observing each possible residue at each position in the LC sequence in the healthy polyclonal repertoire. This procedure allowed “uncommon” residues to be identified, which we hypothesize are important in determining the properties of the LC. We used this approach to prospectively identify positions that frequently harbor uncommon residues in AL-associated LCs. We also evaluated the frequency of individual residue changes that have previously been proposed to be amyloidogenic.

## 2 Methods

### 2.1 Terminology and nomenclature

Throughout this manuscript, we use the IMGT nomenclature and numbering system for LC genes, proteins and residues (47, 57). For each antibody, single germline precursor variable and joining genes (*IGV_L_* and *IGJ_L_*, encompassing both κ and λ gene fragments from the *IGK* and *IGL* loci, respectively) are recombined to yield a variable region (*IGKV-IGKJ* for κ or *IGLV-IGLJ* for λ), encoding the ~110-residue LC V_L_-domain protein. The IMGT numbering system has 127 possible residue positions in LCs, including conserved gaps to accommodate insertions. This numbering system allows direct comparison between the different types of immunoglobulin domains, so several positions such as 58–64 are very rarely occupied by residues within human LCs. “Residue position” always refers to IMGT numbering.

### 2.2 Light chain sequences

This study focuses on the frequency of individual residue changes within LC V_L_-domains associated with AL amyloidosis, MM, or the polyclonal immune repertoire represented by OAS (56). We refer to these sequences as AL, MM and OAS LCs, respectively. The overall workflow is shown in **Supplementary Figure 1**. A “set” of sequences is defined as all the LCs from a specific germline gene and disease or repertoire of origin. Only complete *IGV_L_*-*IGJ_L_* sequences with no ambiguous or missing residues were used for analysis. Sequences with CDR3 insertions longer than the available space in IMGT numbering were also excluded. We did not consider nucleotide sequences or C_L_ domains, since these are not available for all LCs. We refer to “residue changes” rather than “mutations” since only protein sequences were analyzed and to avoid ambiguity between germline and somatic mutations.

In total, 746 AL, 969 MM and 8,047,747 OAS LC sequences were analyzed. AL LCs included monoclonal LC sequences from the AL-Base “AL-PCD” category, comprising all sequences from individuals with a diagnosis of AL amyloidosis, regardless of the underlying hematological malignancy. Note that this selection differed from that used in our previous studies (11, 50), which excluded LC sequences from the AL/MM subcategory. MM LCs included sequences from the “MM” and “SMM” (smoldering MM) subcategories, but excluded those with a diagnosis of light chain deposition disease. Unique OAS LCs were identified from 66 OAS samples derived from healthy adults as previously described (11).

Twenty *IGV_L_* genes (8 *IGKV* and 12 *IGLV*), for which at least five AL LCs were available in the AL-Base data, were analyzed: *IGKV1-12, IGKV1-16, IGKV1-33, IGKV1-39, IGKV1-5, IGKV3-15, IGKV3-20, IGKV4-1*; and *IGLV1-36, IGLV1-40, IGLV1-44, IGLV1-47, IGLV1-51, IGLV2-8, IGLV2-14, IGLV2-23, IGLV3-1, IGLV3-19, IGLV3–21* and *IGLV6-57*. Results in the main body of the manuscript focus on LCs derived from *IGKV1–33* and *IGLV2-14*, which are frequently observed in AL, MM and OAS LCs. Data for other germline genes is shown in **Supplementary Data Sheet 1**.

### 2.3 Alignment

LC nucleotide and protein sequences were assigned to germline precursor genes using the IMGT High-VQuest or DomainGapAlign tools, respectively (58). Paralogous *IGKV* genes from the proximal and distal loci (59) were counted as being from the proximal locus (e.g., LCs assigned to *IGKV1-33* and *IGKV1D-33* were both counted as being derived from *IGKV1-33*). For nucleotide sequences, the translation of the sequence provided by High-VQuest was used. OAS LCs are assigned to a precursor gene as part of the deposition process (56) so these assignments were not reanalyzed. Residues were aligned to the IMGT numbering system using the ANARCI tool (60), which more consistently assigned sequence gaps than other tools for these sequences. The analyses described below are sensitive to misaligned gaps in the IMGT numbering system, which may be misinterpreted as insertions or deletions (indels). There were 71 AL-Base sequences where alignment by ANARCI led to indels, relative to the assigned germline sequence. These were checked manually and 43 alignments were modified to favor residue replacements over indels, so as to maintain the positions of gaps that are present in germline sequences and minimize alignment biases.

### 2.4 Consensus matrices and uncommon residue frequency

For each *IGV_L_* gene, we separately aligned the AL, MM or OAS V_L_ domain sequences using ANARCI (60). Following the approach of Sheng and coworkers (13), we constructed consensus matrices to define the distribution of residues at every position. An example matrix is shown in **Supplementary Figure 2**. The identity of the assigned *IGV_L_* allele and *IGJ_L_* gene were not considered. The number and fraction of sequences with each residue at all 127 IMGT positions was calculated. For each group of sequences, this procedure yielded a 127 × 21 matrix, corresponding to the fraction of each residue, including gaps, at each position. We created a total of 59 matrices, corresponding to AL, MM and OAS LCs from each gene except *IGLV1-36*, for which no MM LCs were present in AL-Base. This approach avoided multiple sequence alignments, which are computationally expensive for large numbers of sequences and are prone to errors around the indels which are frequent in CDR3. The OAS matrices are provided in **Supplementary Table 1**.

To evaluate the residue-wise variability at each position within the matrices, we calculated the Gini coefficient (61), implemented in the R ineq package (62). This is a measure of the inequality of a distribution, which varies between 0 (corresponding to equal fractions of all possible residues) and 1 (a single residue observed in all sequences). To compare the residue-wise differences between sets of sequences, we calculated the Pearson correlation coefficient (ρ) between the columns of the relevant consensus matrices for each pair of residues (ρ = 1 indicates identical distributions of residues).

To compare pairs of consensus matrices, we calculated difference matrices by subtraction. For global comparison between groups of sequences, we calculated the pairwise Euclidian distance between each consensus matrix, which is the square root of the sum of squared differences between each of the positions in the matrices. These distances are equivalent to the total magnitude of the corresponding difference matrix. We subjected the results to hierarchical clustering analysis in R (63) to yield a phylogenetic tree (or dendrogram).

For all AL-Base sequences analyzed, the frequency of each residue within the appropriate OAS consensus matrix was calculated. Based on the distributions of these frequencies, we defined “common” residues as those appearing in ≥ 10% of OAS sequences derived from that germline gene and “uncommon” residues as those appearing in < 10% of OAS sequences. To visualize the frequency in OAS of each residue within a sequence of interest, we plotted the values from the corresponding consensus matrix as a “frequency profile”.

For each group of LCs, the number sequences harboring common and uncommon residues at each position was calculated. For prospective analysis and creation of frequency profile plots, all positions were considered. For previously identified positions, numbers of sequences were counted with both the specific residue change as published, and with any uncommon residue at that position. Residue changes described by Hurle and coworkers (41) were assigned to the germline gene of the LC in which they were originally described. Insertion of proline within CDR3 of an *IGKV1-33*-derived LC was identified as potentially amyloidogenic by Randles and coworkers (64). This is referred to as 95ProIns in the original publication, which uses the Kabat numbering system, and P115PP here using the IMGT numbering system. Identifying these insertions is difficult because of the variable length of CDR3, which can lead to ambiguous alignments. Sequences were identified where two consecutive proline residues occur around IMGT position 115 and at least one residue is inserted into the gap within CDR3, which corresponds to positions 110–113 of the germline *IGKV1-33* sequence.

To compare the frequencies of residue changes within groups of LCs, we calculated the odds ratios (OR), 95% confidence intervals (CI) and associated p-values for each pairwise comparison. These parameters were calculated directly from counts, rather than estimated from a generalized linear model. To avoid division by zero, a correction factor of 0.1 was added to all counts before ORs were calculated. OR significance was calculated directly from the natural logarithms of ORs and their standard errors using the normal distribution, implemented in R (63). Positions with a positive OR for the AL *vs.* OAS comparison, but where only germline residues were observed in AL LCs, were excluded because these were determined to be artifacts caused by the large difference in sample sizes. P-values were corrected for multiple testing using the false discovery rate (FDR) method (65), where FDR < 0.05 was considered statistically significant.

### 2.5 Sequence logos

To visualize residue frequency at each position across the LC sequence, we generated customized sequence logo plots (shown in **Supplementary Data Sheet 1**) using the R package ggseqlogo (66) that differed from the original sequence logo concept (67). Because residue changes were distributed throughout the LC sequence, the information content at each position was dominated by the germline sequence. To highlight non-germline residues, we excluded the most frequent residue from the consensus matrix and created a logo where the height of each letter represented only its frequency in the alignment.

### 2.6 Structural analysis

Crystal structures of germline *IGKV1-33*, *IGLV2-14* and *IGLV6-57* V_L_-domains were available for analysis (PDB entries 2Q20 (43) 6SM1 (44) and 2W0K (68), respectively). In addition, we generated V_L_ domain models for the 20 *IGV_L_* genes studied using the Alphafold 3 server https://alphafoldserver.com/ (69). Protein sequences corresponding to the *01 allele of each *IGKV* or *IGLV* gene and appropriate IGKJ1*01 or IGLJ1*01 gene were submitted using the default parameters. The resulting models had good agreement with the crystal structures (Cα root mean square deviations of 0.476, 0.435 and 0.679 Å for *IGKV1-33*, *IGLV2-14* and *IGLV6-57*, respectively). Secondary structures and solvent-accessible surface areas were calculated using DSSP (70, 71). Residues with < 10% surface exposure, relative to reference peptides (72), were defined as buried and other residues were defined as solvent exposed. Data were mapped onto structures using ChimeraX (73).

### 2.7 Analysis software versions used

Most analysis was carried out using R v 4.2.2 (63) via the RStudio environment (74). The following packages were used: Biostrings v 2.66.0 (75), broom v 1.0.3 (76), cowplot v 1.1.1 (77), furrr v 0.3.1 (78), future v 1.33.0 (79), ggpubr v 0.6.0 (80), ggtree v 3.4.1 (81), ggseqlogo v 0.1 (66), ineq v 0.2-13 (62), msa v 1.30.1 (82), openxlsx v 4.2.8 (83), rstatix v 0.7.2 (84), and Tidyverse v 1.3.2 (85). Local installations of ANARCI (60) and DSSP v 2.1.0 (70, 71) were used on the Boston University Shared Computing Cluster. Alphafold 3 was accessed via the web interface, https://alphafoldserver.com/ (69). ANARCI can also be accessed via a web interface, https://opig.stats.ox.ac.uk/webapps/sabdab-sabpred/sabpred/anarci/.

## 3 Results

### 3.1 Sets of LCs derived from the same germline gene have similar patterns of residue changes

**Figure 1:**
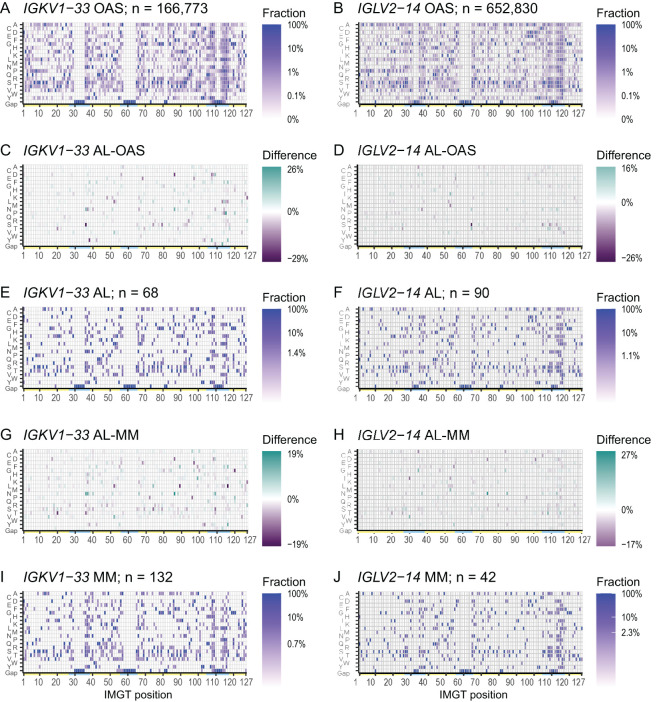
Patterns of residue use are similar between AL, MM and OAS LCs derived from the same *IGV_L_* gene. Heatmaps of the consensus matrices and difference matrices for alignments of AL, MM and OAS LCs derived from *IGKV1-33* (left column) and *IGLV2-14* (right column). Consensus matrix heatmaps **(A, B, E, F, I, J)** show the frequency of each residue, including gaps, in the corresponding multiple sequence alignment. The scale bars show the minimum frequency at which residues were observed. Yellow and cyan lines show the positions of FRs and CDRs, respectively. Difference heatmaps **(C, D, G, H)** are calculated by subtracting each position in the comparator matrix from that in the AL matrix. Residues which are more frequent in AL LCs are shown in green and residues which are less frequent in AL LCs are shown in purple. Heatmaps, sequence logo plots and statistics for 20 *IGV_L_* genes are shown in **Supplementary Data Sheet 1**.

The frequency of mutations varies across LC sequences due to the biased mechanism of somatic hypermutation (13). Within each *IGV_L_* gene, alignments of OAS sequences provide a reference for this variability. We constructed consensus matrices from all OAS LCs for each *IGV_L_* gene, which define the frequency of each residue, including gaps, at all 127 IMGT positions. These matrices are visualized as heat maps in **Figures 1A, B** for *IGKV1-33* and *IGLV2-14*, respectively. Results for other germline genes are shown in **Supplementary Data Sheet 1**. Frequency data for the OAS matrices are provided in **Supplementary Table 1**. The germline residue was present in > 90% of sequences at most positions. However, in positions that vary between alleles or are prone to mutation, the fraction of sequences with the most common residue was lower. These positions are frequent in CDRs but occur throughout the sequences. For the 20 *IGV_L_* genes where at least five AL LCs were available, we generated additional consensus matrices for AL and MM LCs and calculated differences between pairs of matrices. Data for *IGKV1-33* and *IGLV2-14* are shown in **Figure 1**, and data for other *IGV_L_* genes is shown in **Supplementary Data Sheet 1**.

To further evaluate the distribution of residue changes within sets of LCs, we generated sequence logos for AL, MM and OAS LCs, which show the frequency at which each residue was observed along the sequences (**Supplementary Data Sheet 1**). Quantitation of the variation within and between sets of LC sequences showed that the positions that differ between AL and MM LCs are also most variable positions within the sequences (**Supplementary Data Sheet 1**).

To investigate the relative contributions of within-gene and between-gene variability, we measured the pairwise distances between each of the consensus matrices and analyzed the resulting data using hierarchical clustering (**Supplementary Figure 3**). This analysis showed that AL LCs were consistently more closely related to MM and OAS LCs derived from the same gene than to AL LCs derived from other genes.

### 3.2 Uncommon residues are associated with amyloidogenicity in specific sequences

**Figure 2:**
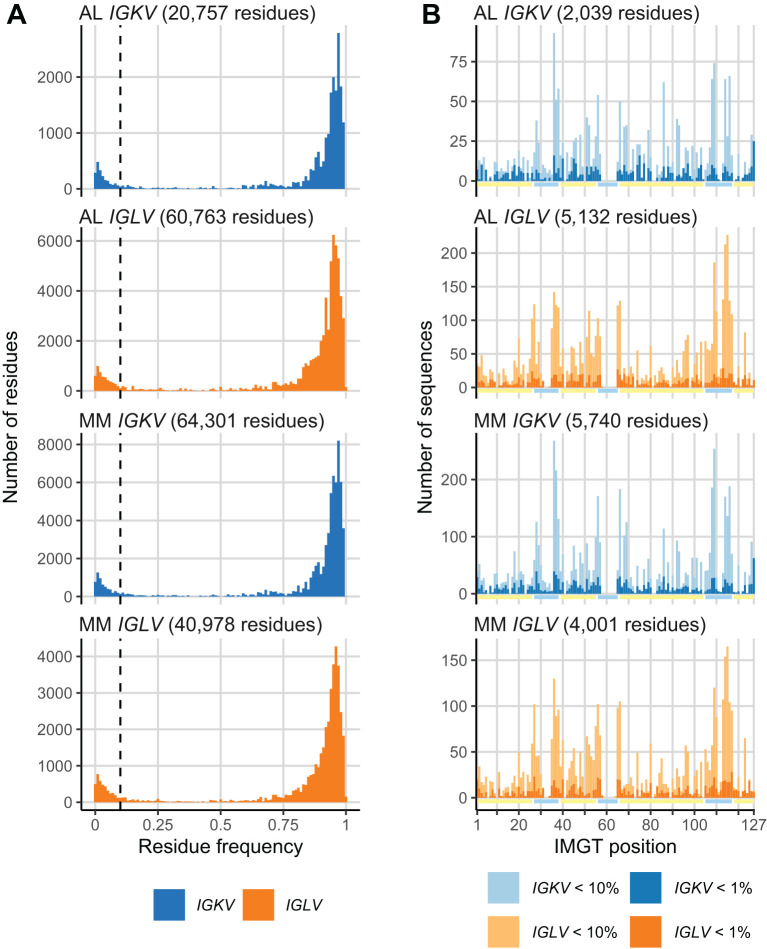
A subset of monoclonal LC residues is uncommon in the OAS repertoire. **(A)** Histograms showing the frequency at which each residue in AL and MM LCs is observed in the OAS consensus matrices. A 10% threshold, used to distinguish common and uncommon residues, is shown as a dashed line. The total number of residues in each set of sequences is shown. **(B)** Distribution of uncommon residues (present in < 10% of OAS sequences) across the sequences of LCs. Residues that occur in < 1% of OAS sequences are shown as darker bars. Yellow and cyan lines show the positions of FRs and CDRs, respectively. The total number of uncommon residues in each set of sequences is shown.

We calculated the frequency at which every residue in AL or MM LC sequences was observed in the OAS consensus matrices. Histograms of these frequencies are shown in **Figure 2A**. Both AL and MM sequences show a bimodal distribution of residue frequencies. Based on these distributions, we defined a residue as “uncommon” if it occurred in < 10% of sequences from the relevant OAS consensus matrix. Residues which occur in ≥ 10% or more of OAS sequences were defined as “common”. Similar proportions of residues were uncommon in both AL and MM LCs (**Figure 2B**) and uncommon residues were observed throughout the LC sequences in both groups (**Figure 2**). We examined the distribution of residues that occur in < 1% of OAS sequences (dark bars in **Figure 2B**), which also occurred throughout the LC sequence.

**Figure 3:**
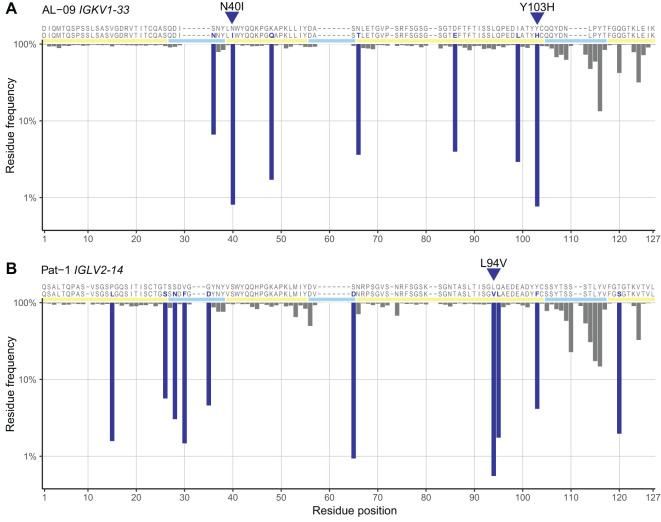
Residue frequency profiles of previously studied AL LCs. The sequences of two LCs, known as AL-09 (43) **(A)** and Pat-1 (44) **(B)** are shown with their assigned germline sequences, aligned to the IMGT numbering system. Residues that differ from the germline sequence are highlighted in dark blue and positions of FRs and CDRs are shown by yellow and cyan lines, respectively. The frequency of each residue and position within the OAS consensus matrices is shown as an inverted bar chart. Residue changes previously identified as destabilizing, which are the least common residues in each sequence, are indicated with blue triangles. The residue changes were originally reported with Kabat numbering (AL-09 N34I and Y87H) or sequential numbering (Pat-1 L81V).

The distribution of residue frequencies was visualized graphically as a “frequency profile” for two amyloidogenic LCs, known as AL-09 and Pat-1, which are derived from the *IGKV1-33* and *IGLV2-14* genes, respectively (43, 44) (**Figure 3**). The frequency of each residue within the OAS repertoire alignments is shown. Baden and coworkers identified N34I and Y87H (N40I and Y103H in IMGT numbering) as destabilizing residue changes in AL-09 *in vitro*, relative to its germline sequence (43). These two residues were observed at lower frequencies within the OAS alignment for *IGKV1-33* (0.81% and 0.77%, respectively) than the rest of the AL-09 sequence. Similarly, Kazman and coworkers identified L81V (IMGT L94V) as highly destabilizing to Pat-1 *in vitro* (44). This residue is the least common of all Pat-1 residues in the OAS alignment for *IGLV2-14* (0.55%).

### 3.3 Residue changes in many positions are associated with AL amyloidosis

**Figure 4:**
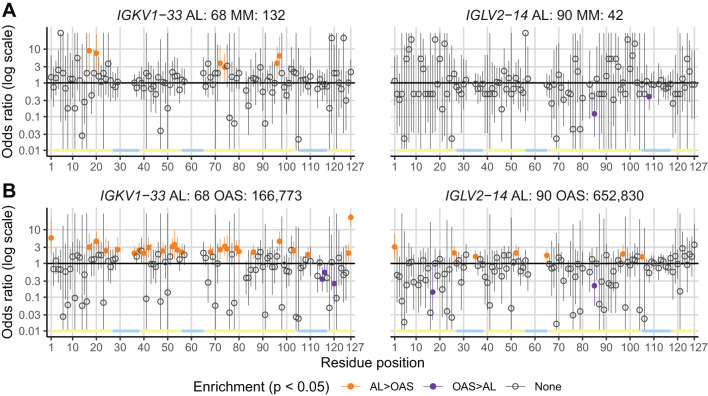
A subset of positions is enriched for uncommon residues in AL *vs.* MM or OAS LCs. Plots of ORs (points) and 95% confidence intervals (lines) for any uncommon residue at each position in LC sequences for the AL *vs.* MM **(A)** and AL *vs.* OAS **(B)** comparisons. Positions where AL LCs are enriched for uncommon residues (p < 0.05 before correction for multiple testing) are highlighted in orange. Positions where AL LCs were less likely to harbor uncommon residues are shown in purple. Positions corresponding to gaps in the germline sequence are not shown. The positions of FRs and CDRs are shown by yellow and cyan lines, respectively. Data for LCs derived from *IGKV1–33* and *IGLV2–14* are shown as examples; figures for other *IGV_L_* genes are shown in **Supplementary Data Sheet 1** and additional data are provided in **Supplementary Table 2**.

To identify residue changes that are enriched or depleted in AL LCs compared with other LCs, we measured the proportion of sequences that harbor an uncommon residue at each position in all AL, MM and OAS LC sequences, segregated according to precursor gene. We calculated ORs and 95% confidence intervals for observing an uncommon residue at each position for AL *vs.* MM and AL *vs.* OAS LCs. Data at each position in LCs derived from *IGKV1-33* and *IGLV2-14* are shown for the AL *vs.* MM (**Figure 4A**) and AL *vs.* OAS (**Figure 4B**) comparisons. Equivalent results for other *IGV_L_* genes are shown in **Supplementary Data Sheet 1**, and details of all positions identified are reported in **Supplementary Table 2**. Without correction for multiple comparisons, six positions in *IGKV1-33*, but no positions in *IGLV2-14*, were significantly enriched for uncommon residues in AL *vs.* MM LCs (p < 0.05, orange symbols). Two positions in *IGLV2-14* harbor uncommon residues less frequently in AL LCs than in MM LCs (p < 0.05, purple symbols). For the comparison between AL and OAS sequences, 26 positions among *IGKV1-33* LCs and 7 positions among *IGLV2-14* LCs were significantly enriched for uncommon residues (p < 0.05, orange symbols). Three positions in *IGKV1-33* LCs and two positions in *IGLV2-14* LCs were under-represented in uncommon residues for the AL *vs.* OAS comparison (p < 0.05, purple symbols).

**Figure 5:**
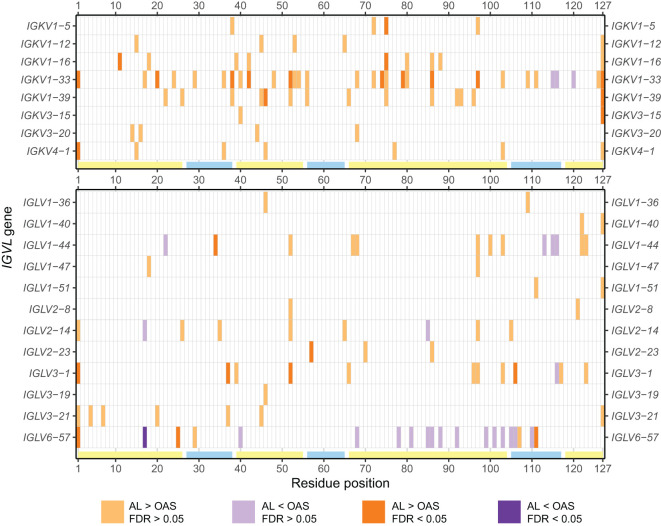
Positions enriched in uncommon residues vary between *IGV_L_* genes. Positions where uncommon residues are significantly more (orange) or less (purple) frequently observed in AL *vs.* OAS LCs are shown for all *IGV_L_* genes examined (p < 0.05). Positions for which significance was retained after correction for multiple testing (FDR < 0.05) are shown as darker shades. The positions of FRs and CDRs are shown by yellow and cyan lines, respectively. Additional information is provided in **Supplementary Table 2**.

Analysis of the 20 precursor genes where at least 5 AL sequences were available showed that the number and location of positions enriched for uncommon residues differ substantially between genes (**Figure 5**; **Supplementary Table 2**). Uncommon residues were over-represented in the AL *vs.* OAS comparison at 62 positions, or 125 gene/position combinations, encompassing all four FRs and three CDRs. Uncommon residues were under-represented in a further 20 positions, or 25 gene/position combinations. Notably, few of these positions were common between precursor genes, with 30 positions observed in only a single gene. The V_L_-domain N- and C-termini were the most frequently enriched positions (6 and 9 genes, respectively), although this may reflect systematic biases due to sequencing strategies. Other positions enriched in uncommon residues in multiple genes were positions 52 (6 genes), 85 (5 genes), 97 (6 genes) and 103 (5 genes). We correlated the variability at each position with its degree of enrichment in the AL *vs.* OAS comparison and observed that uncommon residues were most frequently enriched at positions that were conserved in OAS LCs (**Supplementary Figure 4**). Excluding the N- and C-termini, 85 of 110 positions that were enriched for uncommon residues in AL *vs.* OAS were within FRs (**Figure 5**; **Supplementary Figure 4**).

**Figure 6:**
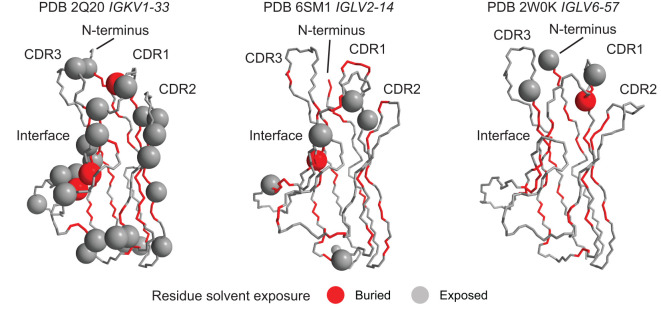
Diverse structural regions are more frequently mutated in AL than OAS LCs. Positions where uncommon residues are more frequent in AL *vs.* OAS LCs (orange panels in **Figure 5**) are shown as spheres on the available crystal structures of isolated V_L_-domains. Other residues are shown as a backbone trace. Residues whose sidechains are not accessible to the solvent are colored red and those with solvent-exposed sidechains are colored gray. The concave β-sheet that forms interface with the heavy chain in antibodies and with a partner LC in homodimers is indicated. Note that not all N-terminal residues are resolved in the structures. Data for other genes are shown in **Supplementary Figure 5**.

We mapped these positions onto the available crystal structures of isolated V_L_-domains corresponding to *IGKV1-33*, *IGLV2-14* and *IGLV6-57* (43, 44, 68) (**Figure 6**). Structural mapping of these data for other *IGV_L_* genes, using computational models of the isolated V_L_ domains, is shown in **Supplementary Figure 5**. Positions enriched for uncommon residues occur in a variety of structural contexts, including both solvent-exposed and buried residues; those in hydrogen-bonded secondary structures and unstructured loops; and residues that interact with the LC’s heavy chain partner. These details are provided in **Supplementary Table 2**. Again, there were no clear patterns of which positions were enriched for AL-associated residues

We next used this data to reexamine positions and residue changes that have previously been identified as associated with AL amyloidosis. A total of 48 positions were studied (**Tables 1**–**3**). Additional information for these positions is provided in **Supplementary Table 3**. We divided the residue changes into three groups, associated with all *IGKV*-derived LCs, specific *IGKV* genes and specific *IGLV* genes.

**Table 1:** Frequency in AL-Base and OAS LC sequences of residue changes previously reported as amyloidogenic among all *IGKV* LCs.

Residue change (references)	Frequency (%)			OR		FDR (p-value)	
	AL	MM	OAS	AL *vs.* MM	AL *vs.* OAS	AL *vs.* MM	AL *vs.* OAS
**X37D** (38, 40)	**11/192** **(5.73)**	**26/590** **(4.41)**	**84,902/4,288,405** **(1.98)**	**1.3**	**2.9**	**0.6** **(0.47)**	**1.10 x 10^-3^** **(5.40 x 10^-4^)**
**X51N** (38, 40)	**18/192** **(9.38)**	**30/595** **(5.04)**	**136,197/4,295,181** **(3.17)**	**1.9**	**3**	**0.1** **(0.045)**	**4.90 x 10^-5^** **(1.00 x 10^-5^)**
X76V (38, 40)	1/192 (0.521)	1/595 (0.168)	31,514/4,295,181 (0.734)	3.1	0.78	0.56 (0.4)	0.8 (0.8)
X83Y (38, 40)	3/192 (1.56)	7/588 (1.19)	133,634/4,264,717 (3.13)	1.3	0.51	0.7 (0.67)	0.3 (0.25)
**X86N** (38, 40, 49)	**27/192** **(14.1)**	**14/595** **(2.35)**	**36,580/4,295,181** **(0.852)**	**6**	**17**	**2.20 x 10^-6^** **(1.60 x 10^-7^)**	**< 10^-20^** **(< 10^-20^)**
**X88N** (38, 40, 49)	**11/192** **(5.73)**	**8/595** **(1.34)**	**24,123/4,295,181** **(0.562)**	**4.2**	**10**	**7.20 x 10^-3^** **(2.10 x 10^-3^)**	**3.40 x 10^-13^** **(4.90 x 10^-14^)**
**ST20X** (38, 40)	**19/192** **(9.9)**	**35/590** **(5.93)**	**192,697/4,288,405** **(4.49)**	**1.7**	**2.2**	**0.14** **(0.084)**	**1.70 x 10^-3^** **(1.00 x 10^-3^)**
I29X (38, 40)	16/146 (11)	47/364 (12.9)	129,106/1,585,731 (8.14)	0.85	1.4	0.7 (0.6)	0.3 (0.25)
**P72X** (38, 40)	**20/192** **(10.4)**	**13/595** **(2.18)**	**183,142/4,295,181** **(4.26)**	**4.8**	**2.5**	**1.20 x 10^-4^** **(2.00 x 10^-5^)**	**4.00 x 10^-4^** **(1.40 x 10^-4^)**
**ST74X** (38, 40)	**18/146** **(12.3)**	**25/359** **(6.96)**	**81,049/1,578,955** **(5.13)**	**1.8**	**2.4**	**0.14** **(0.079)**	**1.10 x 10^-3^** **(4.60 x 10^-4^)**
**R75X** (38, 40)	**15/169** **(8.88)**	**8/590** **(1.36)**	**124,890/4,235,271** **(2.95)**	**6.5**	**3**	**1.20 x 10^-4^** **(2.60 x 10^-5^)**	**1.40 x 10^-4^** **(4.00 x 10^-5^)**
P96X (38, 40)	19/151 (12.6)	22/470 (4.68)	207,638/2,578,061 (8.05)	2.7	1.6	7.20 x 10^-3^ (2.60 x 10^-3^)	0.1 (0.066)
D98X (38, 40)	3/192 (1.56)	2/595 (0.336)	95,042/4,295,181 (2.21)	4.6	0.73	0.14 (0.09)	0.63 (0.58)
Q106X (38, 40)	11/191 (5.76)	39/588 (6.63)	413,149/4,232,213 (9.76)	0.87	0.59	0.7 (0.7)	0.13 (0.093)

Additional information is provided in **Supplementary Table 3**. Residue changes were originally described as gain (e.g., X37D) or loss (e.g., ST20X) of a specific residue, where X represents any residue other than that specified and ST indicates serine or threonine. Only germline *IGKV* genes where the residue change is possible were included in the calculations: genes with the gained residue or without the lost residue were excluded. Residue changes significantly enriched in AL *vs.* OAS LCs (FDR < 0.05) are shown in bold. All residue changes yield an uncommon residue. OR, odds ratio; FDR, false discovery rate.

**Table 2:** Frequency within AL-Base and OAS LC sequences of residue changes previously reported as amyloidogenic for specific *IGKV* germline genes.

*IGV_L_* gene	Residue change (references)	Frequency (%)			OR AL *vs.* MM	OR AL *vs.* OAS	FDR (p-value) AL *vs.* MM	FDR (p-value) AL *vs.* OAS
		AL	MM	OAS				
*KV1-16*	**R75N** (41)	**7/23** **(30.4)**	**0/5** **(0**)	**1,428/59,910** **(2.38)**	**16**	**13**	**0.68** **(0.39)**	**1.10 x 10^-7^** **(1.60 x 10^-8^)**
*KV1-33*	**Y38H** (64, 101)	**6/68** **(8.82)**	**6/132** **(4.55)**	**5,346/166,773** **(3.21)**	**1.9**	**2.8**	**0.68** **(0.26)**	**0.033** **(0.016)**
	Y38S (103)	4/68 (5.88)	13/132 (9.85)	5,538/166,773 (3.32)	0.61	1.8	0.68 (0.39)	0.32 (0.24)
	**N40I** (43, 104, 108)	**6/68** **(8.82)**	**5/132** **(3.79)**	**1,352/166,773** **(0.811)**	**2.3**	**11**	**0.67** **(0.17)**	**1.10 x 10^-7^** **(1.60 x 10^-8^)**
	**P46L** (64, 95)	**2/68** **(2.94)**	**1/132** **(0.758)**	**813/166,773** **(0.487)**	**3.7**	**6.3**	**0.68** **(0.27)**	**0.02** **(0.0086)**
	**K48Q** (43, 105–107)	**4/68** **(5.88)**	**4/132** **(3.03)**	**2,857/166,773** **(1.71)**	**1.9**	**3.5**	**0.68** **(0.35)**	**0.03** **(0.014)**
	**K51N** (104, 108)	**9/68** **(13.2)**	**9/132** **(6.82)**	**7,541/166,773** **(4.52)**	**1.9**	**3**	**0.67** **(0.18)**	**0.0072** **(0.0024)**
	S79R (64, 101, 106)	4/68 (5.88)	3/132 (2.27)	4,356/166,773 (2.61)	2.6	2.3	0.68 (0.22)	0.16 (0.1)
	D86E (64)	2/68 (2.94)	3/132 (2.27)	6,632/166,773 (3.98)	1.3	0.77	0.84 (0.76)	0.75 (0.72)
	**D86H** (64, 101, 106)	**11/68** **(16.2)**	**23/132** **(17.4)**	**12,679/166,773** **(7.6)**	**0.93**	**2.1**	**0.9** **(0.86)**	**0.039** **(0.02)**
	**D86N** (38, 49, 50)	**15/68** **(22.1)**	**4/132** **(3.03)**	**3,161/166,773** **(1.9)**	**7.1**	**12**	**0.0085** **(0.00071)**	**< 10^-20^** **(< 10^-20^)**
	**Y103H** (43, 64)	**3/68** **(4.41)**	**0/132** **(0**)	**1,281/166,773** **(0.768)**	**60**	**5.9**	**0.68** **(0.2)**	**0.0072** **(0.0023)**
	P115PP (64)	6/68 (8.82)	14/132 (10.6)	27,844/166,773 (16.7)	0.84	0.54	0.83 (0.73)	0.2 (0.14)
	Y116Q (64, 101)	1/68 (1.47)	1/132 (0.758)	1,794/166,773 (1.08)	1.9	1.5	0.8 (0.63)	0.72 (0.67)
*KV1-39*	**S28T** (103)	**7/30** **(23.3)**	**9/44** **(20.5)**	**37,334/529,242** **(7.05)**	**1.1**	**3.3**	**0.87** **(0.82)**	**0.012** **(0.005)**
	S36R (103)	2/30 (6.67)	1/44 (2.27)	23,091/529,242 (4.36)	2.8	1.6	0.68 (0.39)	0.59 (0.51)
	**Y55F** (41)	**6/30** **(20**)	**2/44** **(4.55)**	**19,960/529,242** **(3.77)**	**4.3**	**5.4**	**0.51** **(0.085)**	**8.60 x 10^-4^** **(2.10 x 10^-4^)**
*KV1-5*	P46X (40)	1/14 (7.14)	4/102 (3.92)	14,875/375,689 (3.96)	1.9	2	0.76 (0.55)	0.58 (0.5)
	K56DE (40)	2/14 (14.3)	19/102 (18.6)	48,945/375,689 (13)	0.79	1.1	0.84 (0.77)	0.87 (0.87)
*KV4-1*	**L15P** (96, 99)	**2/22** **(9.09)**	**1/28** **(3.57)**	**5,063/510,939** **(0.991)**	**2.4**	**9.5**	**0.72** **(0.47)**	**0.0064** **(0.0019)**
	**N34F** (96, 99)	**1/22** **(4.55)**	**0/28** **(0**)	**245/510,939** **(0.048)**	**14**	**100**	**0.68** **(0.43)**	**1.30 x 10^-5^** **(2.20 x 10^-6^)**
	**K36T** (96, 99)	**1/22** **(4.55)**	**0/28** **(0)**	**2,437/510,939** **(0.477)**	**14**	**10**	**0.68** **(0.43)**	**0.033** **(0.017)**
	P46L (96–100)	1/22 (4.55)	0/28 (0)	3,284/510,939 (0.643)	14	7.7	0.68 (0.43)	0.061 (0.037)
	**Y116P** (96, 99)	**7/22** **(31.8)**	**3/28** **(10.7)**	**43,825/510,939** **(8.58)**	**2.9**	**3.7**	**0.67** **(0.16)**	**0.0097** **(0.0038)**
	Y116Q (96–100)	3/22 (13.6)	1/28 (3.57)	19,195/510,939 (3.76)	3.6	3.7	0.68 (0.27)	0.055 (0.032)

Additional information is provided in **Supplementary Table 3**. Counts refer to the exact residue shown. X represents any residue other than that specified. P115PP refers to insertion of proline into CDR3 (see Methods). DE indicates aspartic acid or glutamic acid. Residue changes significantly enriched (FDR < 0.05) in AL *vs.* OAS LCs are shown in bold. OR, odds ratio; FDR, false discovery rate.

**Table 3:** Frequency within AL-Base and OAS LC sequences of residue changes previously reported as amyloidogenic for specific *IGLV* germline genes.

*IGV_L_* gene	Residue change (refs)	Frequency (%)			OR AL *vs.* MM	OR AL *vs.* OAS	FDR (p-value) AL *vs.* MM	FDR (p-value) AL *vs.* OAS
		AL	MM	OAS				
*LV1-44*	A100T (41)	1/77 (1.3)	3/42 (7.14)	3,158/338,214 (0.934)	0.19	1.5	0.67 (0.15)	0.72 (0.66)
*LV1-51*	G84D (41)	0/32 (0)	0/19 (0)	9,150/186,959 (4.89)	0.6	0.063	0.92 (0.91)	0.47 (0.38)
*LV2-14*	L94V (44)	1/90 (1.11)	0/42 (0)	3,617/652,830 (0.554)	5.1	2.2	0.8 (0.62)	0.49 (0.41)
*LV3-19*	G56R (109–111)	1/17 (5.88)	0/16 (0)	1,118/151,676 (0.737)	10	8.7	0.72 (0.48)	0.051 (0.028)
	**G113A** (109–111)	**2/17** **(11.8)**	**1/16** **(6.25)**	**2,012/151,676** **(1.33)**	**1.8**	**9.2**	**0.8** **(0.63)**	**0.0073** **(0.0026)**
*LV6-57*	F2X (89)	4/137 (2.92)	3/9 (33.3)	4,844/90,110 (5.38)	0.089	0.56	0.038 (0.0048)	0.32 (0.24)
	**R25G** (46, 86–89)	**33/137** **(24.1)**	**0/9** **(0)**	**5,958/90,110** **(6.61)**	**22**	**3.6**	**0.68** **(0.33)**	**9.30 x 10^-10^** **(9.70 x 10^-11^)**
	N37T (102)	5/137 (3.65)	0/9 (0)	7,587/90,110 (8.42)	3.4	0.44	0.83 (0.7)	0.11 (0.07)
	G70E (41)	2/137 (1.46)	0/9 (0)	2,798/90,110 (3.11)	1.4	0.49	0.92 (0.92)	0.39 (0.31)

Additional information is provided in **Supplementary Table 3**. Counts refer to the exact residue shown. X represents any residue other than that specified. Residue changes significantly enriched (FDR < 0.05) in AL *vs.* OAS LCs are shown in bold. OR, odds ratio; FDR, false discovery rate.

**Figure 7:**
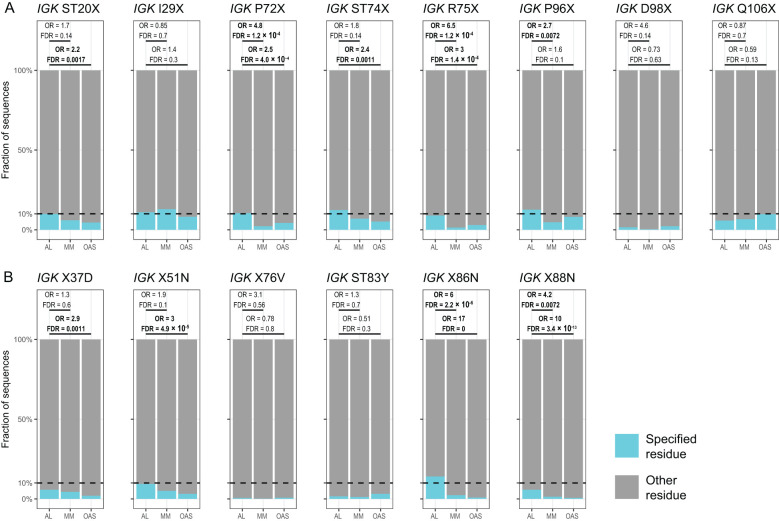
Enrichment of previously identified amyloidogenic residue changes in *IGKV*-derived LCs. Additional information is shown in **Table 1** and **Supplementary Table 3**. The fraction of AL, MM and OAS LCs harboring each residue is shown in cyan. ST indicates serine or threonine; X indicates any residue other than the specified residue. ORs and significance are indicated for the AL *vs.* MM (upper line) and AL *vs.* OAS (lower line) comparisons. Significant comparisons (FDR < 0.05) are highlighted in bold. A 10% threshold, used to define uncommon residues, is shown as a dashed line. **(A)** Residue changes identified as replacement of the germline residue by any residue, calculated for all *IGKV* genes where the indicated residue is present. **(B)** Residue changes identified as acquisition of the specified residue from any germline residue.

**Table 1** and **Figure 7** show residue changes proposed to be associated with amyloidosis in all *IGKV*-derived LCs. All changes yielded an uncommon residue at the specified position. Eight of 14 residue changes were significantly enriched in AL *vs.* OAS LCs. Five residue changes were significantly enriched in AL *vs.* MM LCs, of which four were also significantly enriched compared to OAS LCs. However, the frequency of each residue change in AL LCs was low, accounting for at most 12% of *IGKV*-derived AL LCs. The most significantly enriched residue change in this group was gain of an asparagine residue at position 86 (X86N), which was previously identified as a site for N-glycosylation (38, 49, 50).

**Figure 8:**
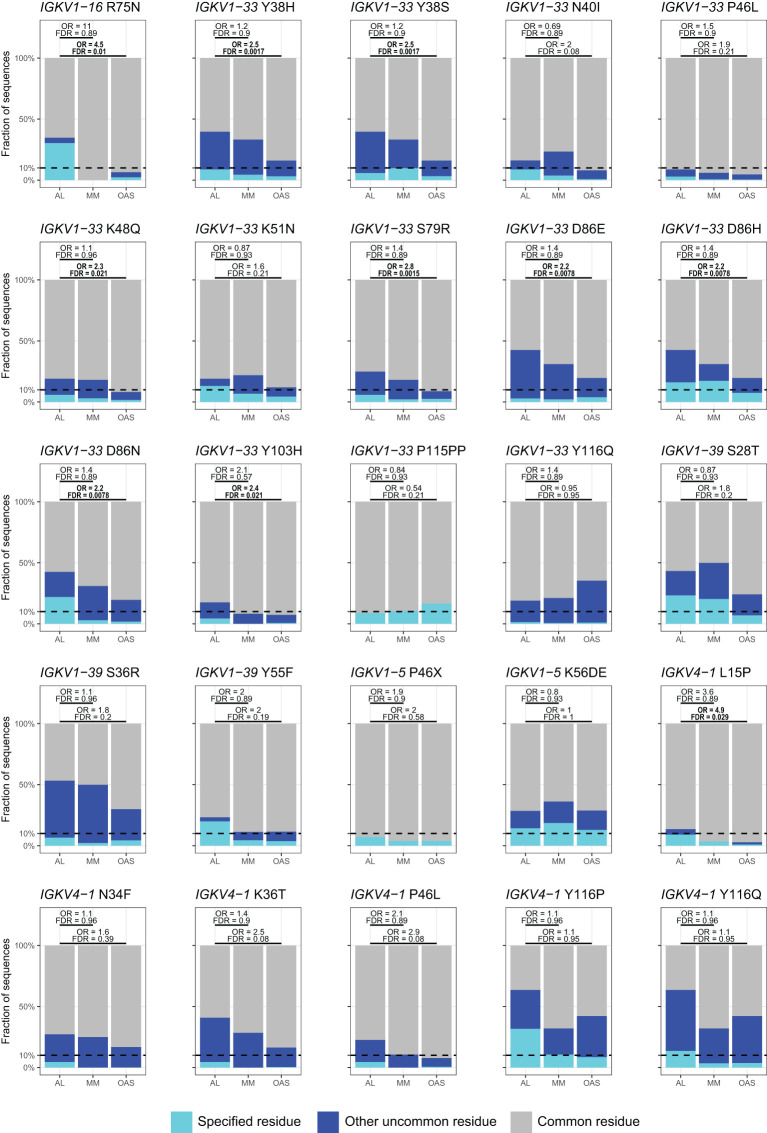
Enrichment of previously identified amyloidogenic residue changes in LCs derived from specific *IGKV* genes. Additional information is shown in **Table 2** and **Supplementary Table 3**. The fraction of AL, MM and OAS LCs harboring the specified residue change is shown in cyan and the fraction harboring any other uncommon residue is shown in dark blue. P115PP indicates insertion of a second proline residue next to the germline-encoded proline within CDR3 of *IGKV1-33*. ORs and significance, calculated for all uncommon residues at each position (**Supplementary File 1**), are indicated for the AL *vs.* MM (upper line) and AL *vs.* OAS (lower line) comparisons. Significant comparisons (FDR < 0.05) are highlighted in bold. A 10% threshold, used to define uncommon residues, is shown as a dashed line.

**Figure 9:**
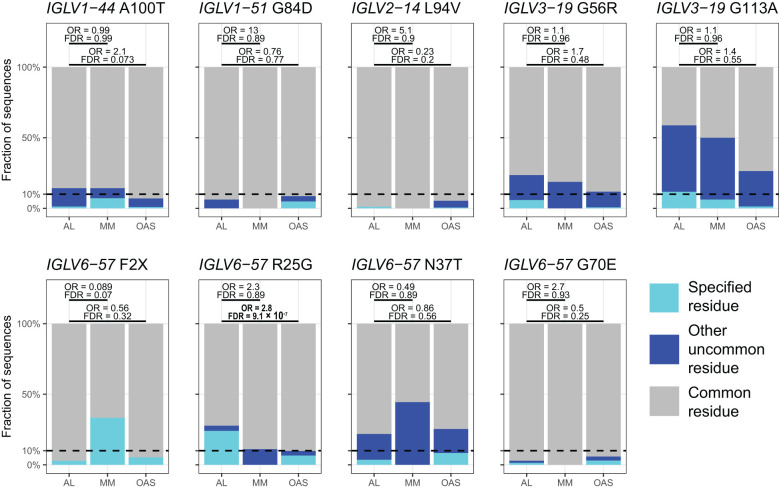
Enrichment of previously identified amyloidogenic residue changes in LCs derived from specific *IGLV* genes. Additional information is shown in **Table 3** and **Supplementary Table 3**. The fraction of AL, MM and OAS LCs harboring the specified residue change is shown in cyan, while the fraction harboring any other uncommon residue is shown in dark blue. ORs and significance, calculated for all uncommon residues at each position (**Supplementary Table 3**), are indicated for the AL *vs.* MM (upper line) and AL *vs.* OAS (lower line) comparisons. Significant comparisons (FDR < 0.05) are highlighted in bold. A 10% threshold, used to define uncommon residues, is shown as a dashed line.

**Table 2** and **Figure 8** show residue changes identified in specific *IGKV* genes, and **Table 3** and **Figure 9** show residue changes identified in specific *IGLV* genes. The tables show the frequency of the specified substitution and the figures show the frequencies of both the specified residue and all uncommon residues. Equivalent calculations for any uncommon residue at each position are provided in **Supplementary File 1**. Of the 34 residue changes, 32 yielded uncommon residues at that position. Seventeen of the 25 previously identified residue changes in *IGKV* genes are significantly enriched (OR > 1, FDR < 0.05) among AL *vs.* OAS LCs (**Table 2**). Any uncommon residue was significantly more frequent in AL than OAS LCs at 10 positions (**Figure 8**). Three residue changes in *IGLV* genes were significantly enriched among AL *vs.* OAS LCs (**Table 2**), but uncommon residues were only significantly enriched at position 25 of *IGLV6-57* (**Figure 9**). No positions were enriched for specified or uncommon residues in the AL *vs.* MM comparison.

### 3.4 Glycine at position 25 of *IGLV6–57* LCs is associated with AL amyloidosis

Our prospective analysis identified position 25 in *IGLV6-57* as the site of an amyloid-associated residue change, in agreement with previous studies (46, 86–90). This residue change is associated with a germline-encoded polymorphism, rather than a somatic hypermutation: the IGLV6-57 *01, *03 and *04 alleles encode an arginine residue (R25), whereas the IGLV6-57*02 allele has a C>G nucleotide transversion that encodes a glycine residue at position 25 (G25). The presence of G25 has been proposed to be associated with AL amyloidosis (46, 86–90), which is supported by our analysis (**Table 3**; **Figure 9**). R25 forms a cation-π interaction that stabilizes the native V_L_ domain, while G25 is destabilizing in the context of the V_L_-domain and the full-length LC (46, 91). We therefore investigated the distribution of residues at position 25 in AL, MM and OAS LC sequences derived from *IGLV6-57* as a function of the assigned allele.

Among 137 AL LCs, 33 were G25 (24%), 99 were R25 (72%) and five had other residues at this position (**Supplementary Figure 6**). None of the 9 *IGLV6-57*-derived MM LCs, and 5,958 of 90,110 *IGLV6-57*-derived OAS LCs harbored G25 (6.6%). The OR for non-argenine residues between AL and MM was 2.32 (p = 0.42). The ORs between AL and OAS were 2.81 for non-argenine residues (p = 6.17 × 10^-8^) and 3.65 for glycine residues (p = 9.67 × 10^-11^). All AL LCs with G25 were assigned to the IGLV6-57*02 allele (**Supplementary Figure 6**). Of the 36 IGLV6-57*02 AL LC sequences, 33 harbored a glycine at position 25, two had alanine and one had a gap when aligned to IMGT numbering. The single MM LC assigned to IGLV6-57*02 had alanine at position 25. OAS LCs were assigned only to the *01 and *02 alleles and all sequences with G25 were derived from the *02 allele.

We repeated this analysis on 46 positions within 15 *IGV_L_* genes where there were residue differences between alleles (**Supplementary Figure 7**). The five other *IGV_L_* genes studied had either a single allele or no residue differences between the alleles. In many cases, there was substantial heterogeneity at these positions. However, none of these positions showed a significant difference between AL and MM, other than *IGLV6-57* R25G, after correction for multiple testing.

## 4 Discussion

Using monoclonal LC sequences from AL-Base and polyclonal sequences from OAS, we have developed a new framework for analyzing potentially amyloidogenic LC sequences. Consensus matrices derived from the polyclonal antibody repertoire provide a germline gene-specific reference frequency for each LC residue at each position (**Figure 1**; **Supplementary Data Sheet 1** and **Supplementary Table 1**). The consensus matrices allowed the variability at each position to be assessed (**Figure 2**), thereby identifying “uncommon” residues that may have important roles in amyloidogenicity (**Figure 3**). We calculated the relative likelihood of observing uncommon residues at each position in LCs derived from 20 *IGV_L_* genes (**Figure 4** and **Supplementary Data Sheet 1**), observing that each gene has a unique set of residues that are more frequently mutated in AL than in MM or OAS LCs (**Figure 5**; **Supplementary Table 2**). These residues are distributed throughout the LC sequences and structures and do not generally correspond to regions with the most variable residues (**Figures 5**, **6**; **Supplementary Data**). We observed that residue changes that have previously been identified as amyloidogenic were present in only a minority of AL LC sequences, even when enriched relative to MM or OAS LCs (**Figures 7**–**9**; **Tables 1**–**3**; **Supplementary Table 3**). These residue changes yielded an uncommon residue in 46 of 48 cases (95.8%), supporting our hypothesis that uncommon residues are associated with amyloidogenicity.

Our data provide new insights into the contributions to LC amyloidogenicity of precursor *IGV_L_* gene identity and somatic hypermutation. Identifying LCs that could deposit as amyloid in humans is an important goal, both to elucidate molecular mechanisms of disease and to aid diagnosis. Identifying and evaluating the sequence of a clonal LC associated with pre-symptomatic PCD could allow earlier intervention, which is critical for patients’ survival (2, 92). A diagnostic tool would need to distinguish between monoclonal LCs secreted by proliferative plasma cells, so MM LCs, which circulate at high levels but are not reported as forming amyloid are an appropriate control. Several computational approaches have been published (38, 51–54), but due to the rarity of amyloidosis these tools are insufficiently selective to be used clinically (11).

Although precursor gene use is an important contributor to amyloid propensity, most AL clones express LCs derived from a gene that is common in the healthy repertoire and MM (11). Therefore, the specific pattern of somatic hypermutations must play a role in pathology. Sequence conservation is associated with protein structure and stability (93, 94), and many studies have looked for residue changes that are associated with amyloidosis (10, 38–46, 49, 64, 68, 86–89, 95–111). However, because the likelihood of each residue change depends on the underlying DNA sequence of each germline gene (13), the distribution of residues at each position differs between genes (**Figure 1**) and the positions of amyloid-associated residue changes are distinct for each gene (**Figure 5**). Consistent with this, the distance between consensus matrices corresponding to AL and MM LCs from the same gene is less than that between AL LCs from closely related genes (**Supplementary Figure 3**). These factors hamper locus-wide comparisons between *IGV_L_* genes (38, 40, 112, 113). Therefore, any evaluation of residue changes needs to be within sequences derived from the same *IGVL* gene. Polymorphism between *IGV_L_* alleles further blurs the distinction between wild-type and variant sequences, so that even the word “mutation” is often used ambiguously in the context of antibodies. This problem is compounded by the limited annotation of human immunoglobulin alleles (48).

Our approach of defining uncommon residues avoids these problems and allows a focus on positions which are differentially mutated between sets of LCs, rather than highly variable in healthy LCs. For many amyloidogenic proteins, single residue changes are sufficient to cause disease and are readily identified (114), but this is not the case for LCs (11). We suggest that defining common and uncommon residues at each position relative to a consensus matrix, rather than wild-type and variant residues, is a more useful framework for considering LC sequence diversity in AL amyloidosis. Because the frequency at which a residue is observed in the OAS LC sequences may report on its compatibility within native antibodies (13, 93), the least common residues in a LC sequence may be particularly disruptive. The frequency profiles shown in **Figure 3** may be a useful tool to simplify the mutational analysis of LCs, by allowing prioritization of residue changes for detailed study. Several studies have systematically mutated residues to identify changes that promote unfolding or aggregation, either from the inferred germline residue to the AL-associated residue, or vice versa (41, 43, 44). However, the time and effort required to exhaustively test each individual residue change has limited the number of LCs studied. For the two examples shown, we observed that the residue changes identified by biophysical analysis (43, 44) yield the most uncommon residues in the sequence. We hypothesize that such residue changes have an outsized effect on the behavior of the LC. However, uncommon residues are similarly frequent among AL and MM LCs, and distributed throughout the sequence (**Figure 2**). Therefore, the presence of an uncommon residue is neither necessary nor sufficient for amyloidogenesis and these potentially amyloidogenic residue changes still need to be investigated. Studying positions that are enriched in uncommon residues in multiple germline genes may reveal shared mechanisms of amyloidogenicity. For example, replacements of a surface-exposed leucine at position 52 are enriched in AL LCs derived from the *IGKV1-33, IGKV1-39, IGLV1-44, IGLV2-14, IGLV2-8* and *IGLV3-1* genes (**Figure 5**; **Supplementary Data 3**). Such replacements could alter the structure and interactions of both the native LC and its amyloid fibrils. The consensus matrices that we constructed for each *IGV_L_* gene capture the likelihood of every possible residue change, allowing an estimate of how well the protein can tolerate changes at that position. The large number of available sequences in OAS allow highly specific residue frequencies to be used to evaluate residue changes, rather than relying on generic substitution matrices such BLOSUM (115). There is incomplete overlap between the positions where specific residues are enriched in AL LCs and those where any uncommon residue is enriched, due to the differences in residue frequencies between the sets of LCs (**Figures 8**, **9**). These differences likely reflect the distribution of residues at each position. Due to the large number of potential residue changes, we did not attempt to predict the effects of each change or classify residues by physicochemical properties. However, structural hypotheses about the potential effect of different classes of residue changes could be evaluated based on examination of the appropriate column of the consensus matrix.

Several studies have sought mutational “hotspots” or regions where AL LCs are frequently mutated to increase their amyloidogenicity (112, 113, 116, 117). Our data instead support a model where many individual residue changes can promote amyloidogenicity, but specific recurrent residue changes account for only a small fraction of amyloidogenic sequences (**Figures 7**–**9**). Importantly, the residues that are most frequently mutated in AL LCs (primarily CDR1 and CDR3, **Figure 1**) are not enriched in uncommon residues when compared to MM or OAS LCs (**Figures 5**, **6**; **Supplementary Figure 4**). This counter-intuitive observation is due to the biased, gene-specific distribution of somatic hypermutations during affinity maturation (13). These results are consistent with previous observations that a limited number of residue changes have substantial effects on the properties of LCs, perhaps analogous to “driver” and “passenger” mutations associated with cancer (118). Examples include R25G in *IGLV6-57* (46) and the X86N and X88N residue changes in *IGKV1* LCs that create an N-glycosylation site (49). Such a model implies that the effects of each residue change must be determined to predict the risk of amyloidosis associated with a new LC sequence. Residue changes can alter the structure of the native V_L_-domain, the amyloid fibril and the intermediate states between these endpoints, as well as interactions of these states with other tissue factors (119). However, few LC proteins have been subject to the mutational analysis needed to determine the effects of an uncommon residue on the behavior of the protein. An advantage of sequence-based approaches to predicting amyloidogenicity is that they are agnostic to mechanism and can identify residue changes that modulate different processes.

This study is limited by the number and diversity of monoclonal AL and MM LCs available, which limits the statistical power of our analyses. In particular, the low numbers of monoclonal LCs derived from several genes limited our ability to detect positions enriched in uncommon residues. To compensate for this, we have reported positions where significance was lost after correction for multiple testing to highlight potentially relevant positions, acknowledging that this increases the risk of including false positives. We anticipate that additional monoclonal LC sequences will become available, allowing validation of our results on independent data. The residue frequencies for OAS LCs, particularly polymorphisms such as *IGLV6-57* R25G, depend on the genotypes of the individuals whose immune repertoires were sequenced, which we did not attempt to infer. We did not consider the roles of the C_L_-domain or the LC’s heavy chain partner due to the lack of available sequences. Furthermore, it remains unclear whether MM and OAS LCs can truly be considered non-amyloidogenic, since these proteins can be induced to aggregate *in vitro* and their ability to form amyloid in patients is unknown. We assume that because amyloidosis is rare even in the context of a PCD, most normal repertoire LCs would not cause disease if overexpressed, but this is not known.

Amyloid formation by antibody LCs remains poorly understood. Our data clearly show that there are no individual residue changes that unambiguously lead to amyloidosis and provide an important resource for further investigation of LC amyloid propensity. By defining the mutational landscape of all LCs, we enable a more quantitative approach to both computational and laboratory studies of sequences.

## Data Availability

Publicly available datasets were analyzed in this study. This data can be found here: https://wwwapp.bumc.bu.edu/BEDAC_ALBase/ and https://opig.stats.ox.ac.uk/webapps/oas/.
