# Supplementary material for: Clone-specific residue changes at multiple positions are associated with amyloid formation by antibody light chains
Source: Front Immunol. 2025 Aug 1;16:1622207. doi: 10.3389/fimmu.2025.1622207 (PMC12375997; doi:10.3389/fimmu.2025.1622207)
Supplement: Supplementary file 1 [file DataSheet1.pdf]

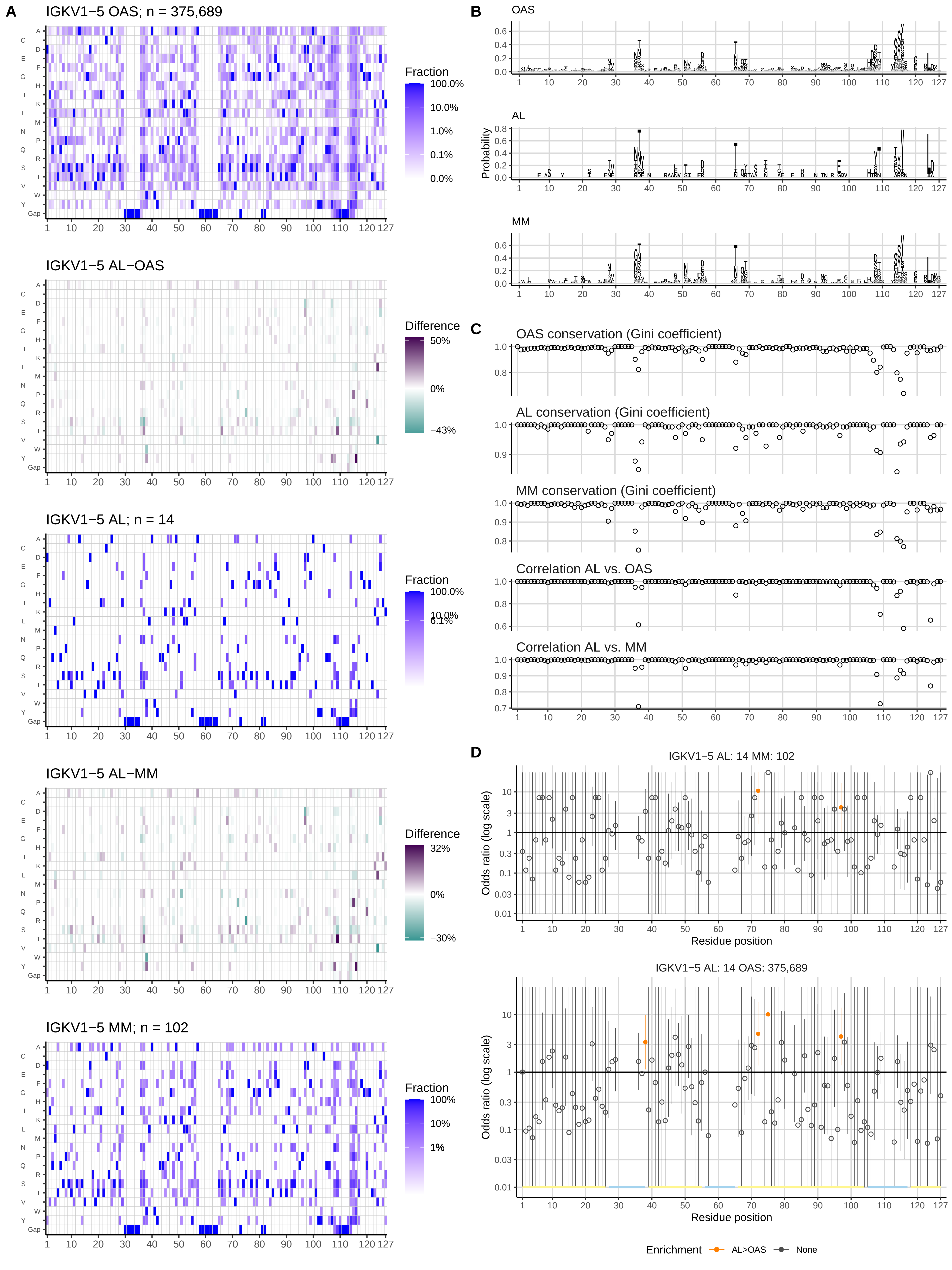
A
IGKV1−5 OAS; n = 375,689
Fraction
100.0%
10.0%
1.0%
0.1%
0.0%
IGKV1−5 AL−OAS
Difference
50%
0%
−43%
IGKV1−5 AL; n = 14
Fraction
100.0%
10.0%
6.1%
IGKV1−5 AL−MM
Difference
32%
0%
−30%
IGKV1−5 MM; n = 102
Fraction
100%
10%
1%
A
C
D
E
F
G
H
I
K
L
M
N
P
Q
R
S
T
V
W
Y
Gap
1 10 20 30 40 50 60 70 80 90 100 110 120 127
B
OAS
AL
MM
Probability
C
OAS conservation (Gini coefficient)
AL conservation (Gini coefficient)
MM conservation (Gini coefficient)
Correlation AL vs. OAS
Correlation AL vs. MM
D
IGKV1−5 AL: 14 MM: 102
IGKV1−5 AL: 14 OAS: 375,689
Odds ratio (log scale)
Residue position
Enrichment
AL>OAS
None

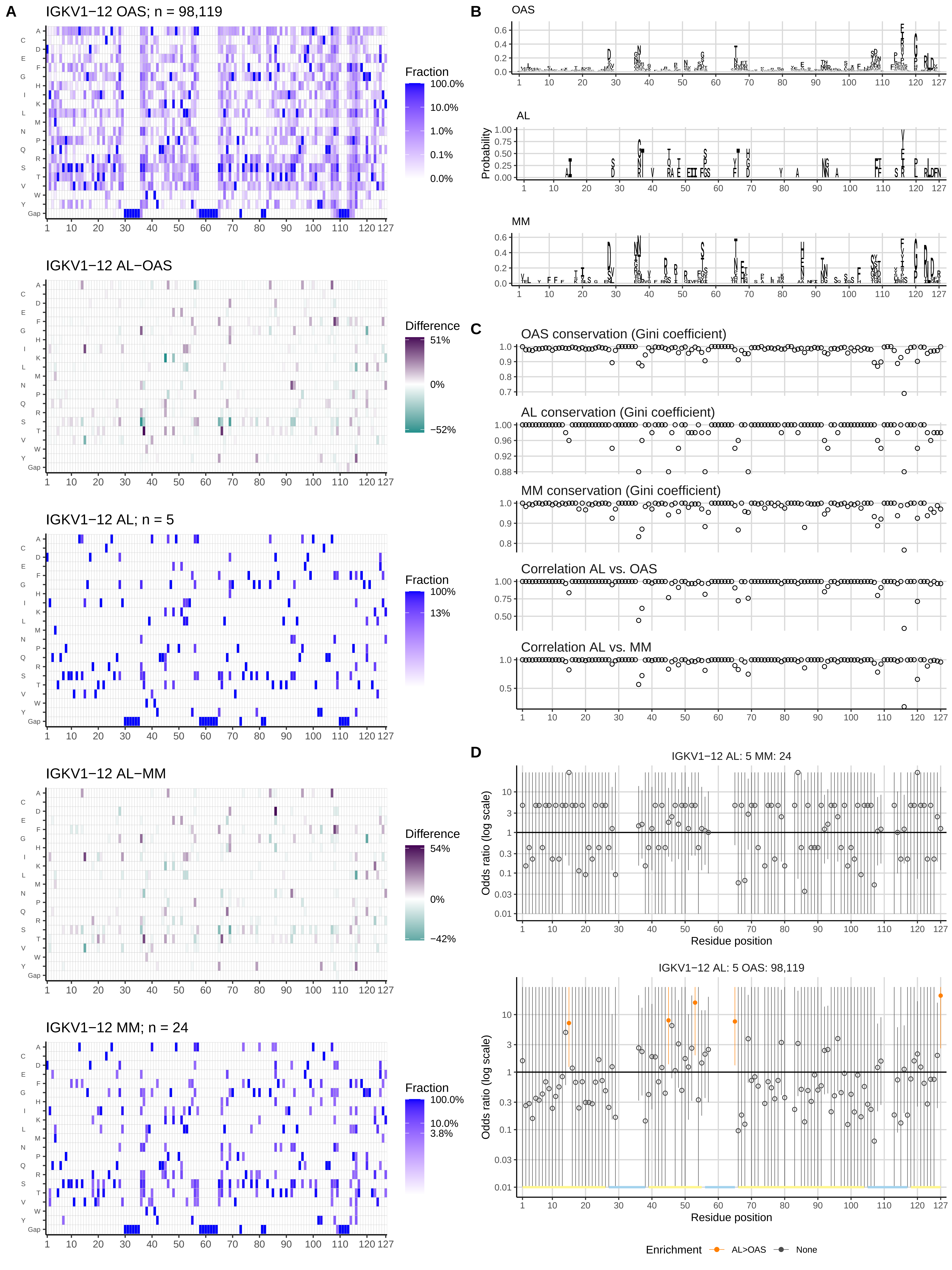
A
IGKV1−12 OAS; n = 98,119
Fraction
100.0%
10.0%
1.0%
0.1%
0.0%
IGKV1−12 AL−OAS
Difference
51%
0%
−52%
IGKV1−12 AL; n = 5
Fraction
100%
13%
IGKV1−12 AL−MM
Difference
54%
0%
−42%
IGKV1−12 MM; n = 24
Fraction
100.0%
10.0%
3.8%
B
OAS
AL
MM
Probability
C
OAS conservation (Gini coefficient)
AL conservation (Gini coefficient)
MM conservation (Gini coefficient)
Correlation AL vs. OAS
Correlation AL vs. MM
D
IGKV1−12 AL: 5 MM: 24
Odds ratio (log scale)
Residue position
IGKV1−12 AL: 5 OAS: 98,119
Odds ratio (log scale)
Residue position
Enrichment
AL>OAS
None

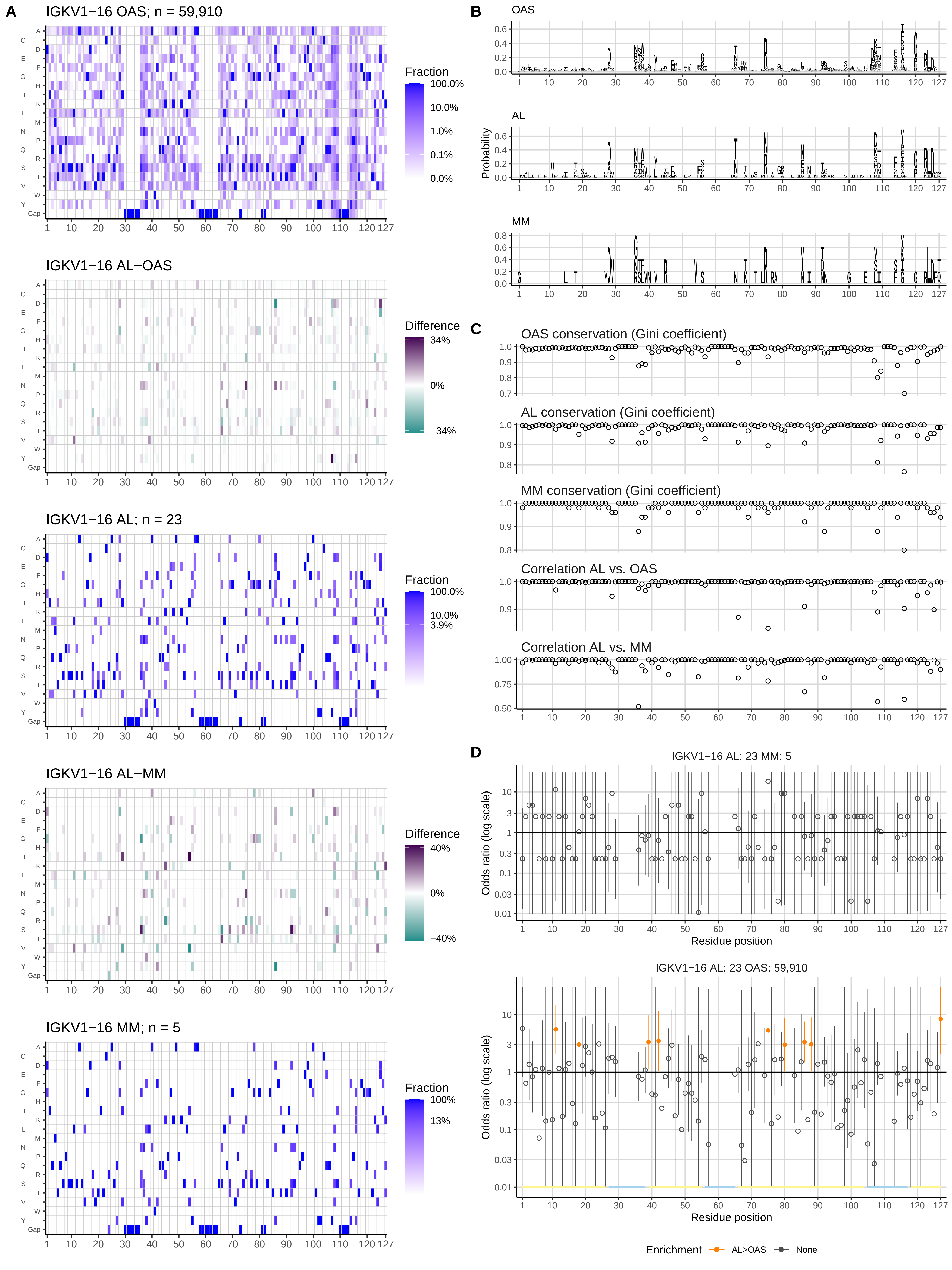
A
IGKV1−16 OAS; n = 59,910
Fraction
100.0%
10.0%
1.0%
0.1%
0.0%
IGKV1−16 AL−OAS
Difference
34%
0%
−34%
IGKV1−16 AL; n = 23
Fraction
100.0%
10.0%
3.9%
IGKV1−16 AL−MM
Difference
40%
0%
−40%
IGKV1−16 MM; n = 5
Fraction
100%
13%
B
OAS
AL
MM
Probability
C
OAS conservation (Gini coefficient)
AL conservation (Gini coefficient)
MM conservation (Gini coefficient)
Correlation AL vs. OAS
Correlation AL vs. MM
D
IGKV1−16 AL: 23 MM: 5
Odds ratio (log scale)
Residue position
IGKV1−16 AL: 23 OAS: 59,910
Odds ratio (log scale)
Residue position
Enrichment
AL>OAS
None

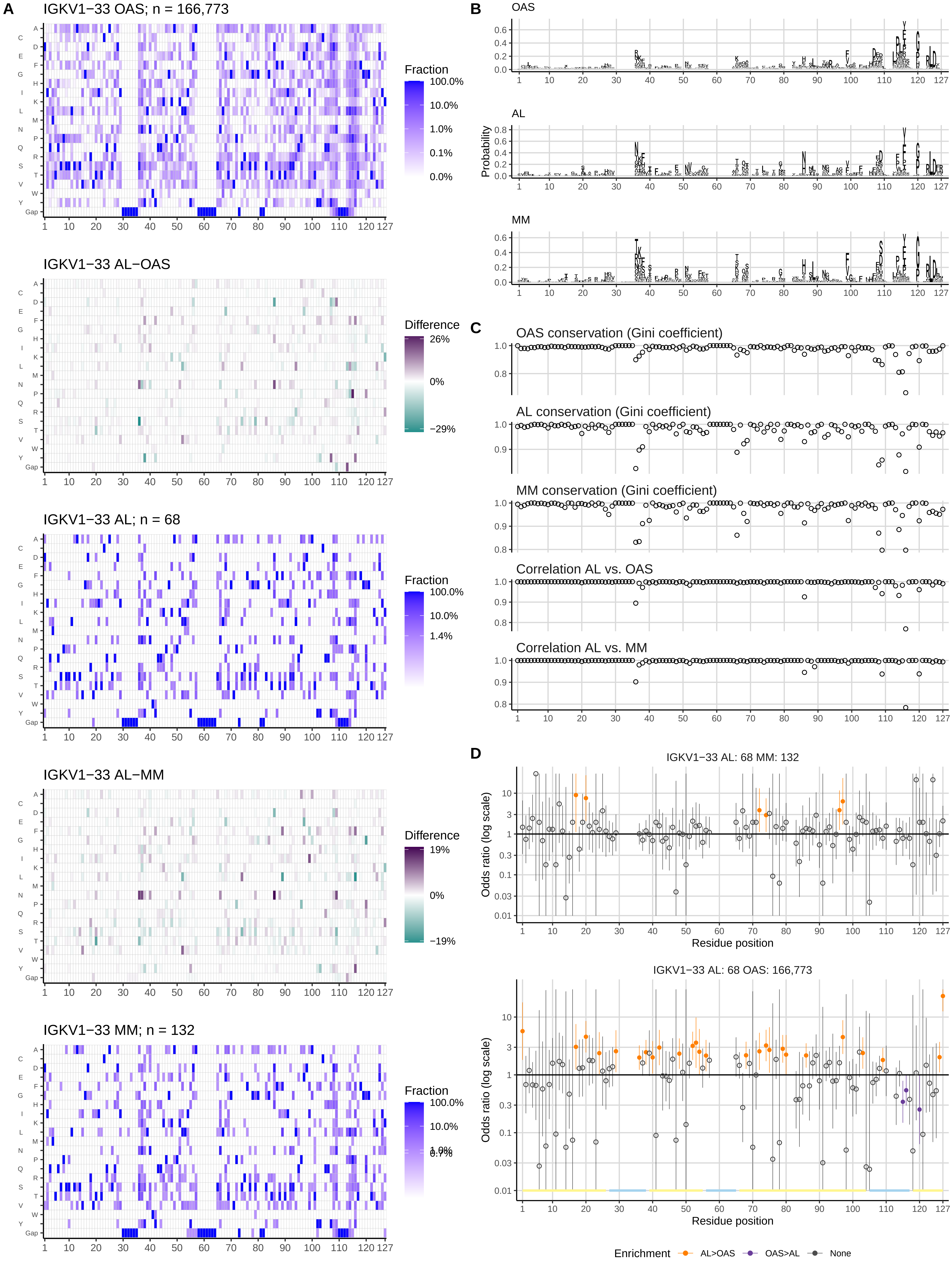
A
IGKV1−33 OAS; n = 166,773
Fraction
100.0%
10.0%
1.0%
0.1%
0.0%
IGKV1−33 AL−OAS
Difference
26%
0%
−29%
IGKV1−33 AL; n = 68
Fraction
100.0%
10.0%
1.4%
IGKV1−33 AL−MM
Difference
19%
0%
−19%
IGKV1−33 MM; n = 132
Fraction
100.0%
10.0%
1.0%
0.7%
A C D E F G H I K L M N P Q R S T V W Y Gap
1 10 20 30 40 50 60 70 80 90 100 110 120 127
B
OAS
AL
MM
Probability
C
OAS conservation (Gini coefficient)
AL conservation (Gini coefficient)
MM conservation (Gini coefficient)
Correlation AL vs. OAS
Correlation AL vs. MM
D
IGKV1−33 AL: 68 MM: 132
IGKV1−33 AL: 68 OAS: 166,773
Odds ratio (log scale)
Residue position
Enrichment
AL>OAS
OAS>AL
None

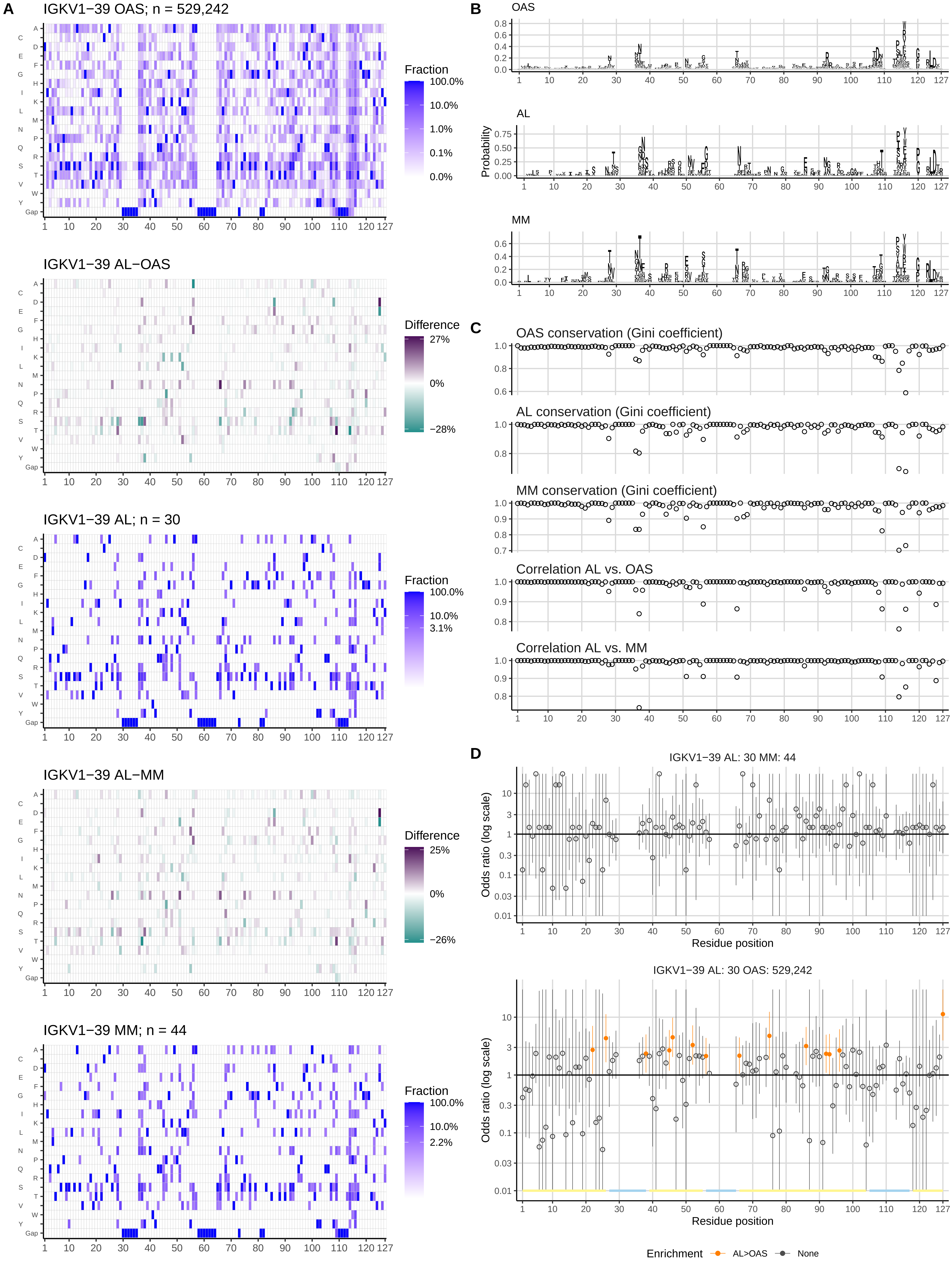
A
IGKV1−39 OAS; n = 529,242
Fraction
100.0%
10.0%
1.0%
0.1%
0.0%
IGKV1−39 AL−OAS
Difference
27%
0%
−28%
IGKV1−39 AL; n = 30
Fraction
100.0%
10.0%
3.1%
IGKV1−39 AL−MM
Difference
25%
0%
−26%
IGKV1−39 MM; n = 44
Fraction
100.0%
10.0%
2.2%
A C D E F G H I K L M N P Q R S T V W Y Gap
1 10 20 30 40 50 60 70 80 90 100 110 120 127
B
OAS
AL
MM
Probability
C
OAS conservation (Gini coefficient)
AL conservation (Gini coefficient)
MM conservation (Gini coefficient)
Correlation AL vs. OAS
Correlation AL vs. MM
D
IGKV1−39 AL: 30 MM: 44
IGKV1−39 AL: 30 OAS: 529,242
Odds ratio (log scale)
Residue position
Enrichment
AL>OAS
None

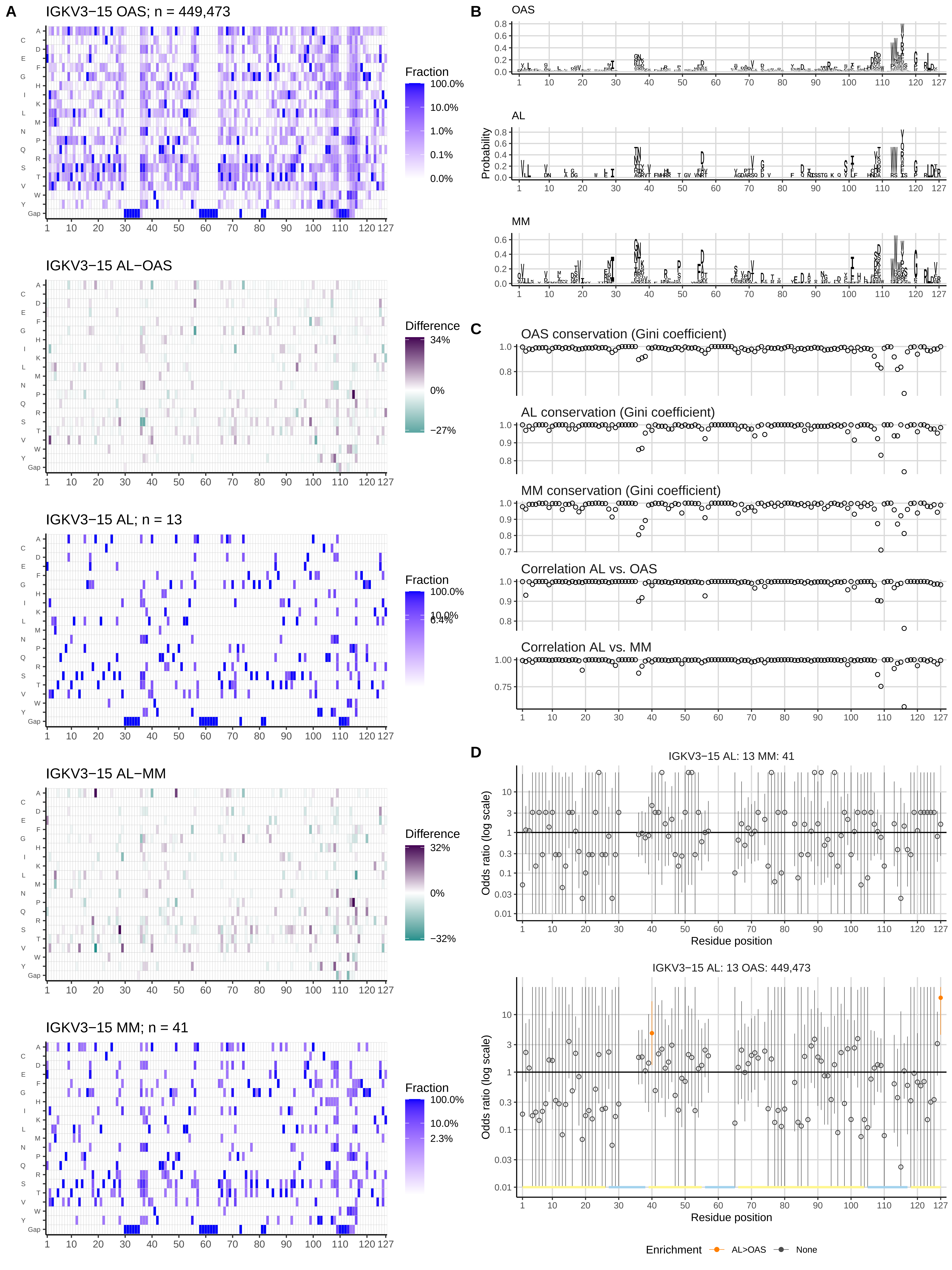

A
IGKV3−15 OAS; n = 449,473
Fraction
100.0%
10.0%
1.0%
0.1%
0.0%
IGKV3−15 AL−OAS
Difference
34%
0%
−27%
IGKV3−15 AL; n = 13
Fraction
100.0%
10.0%
6.4%
IGKV3−15 AL−MM
Difference
32%
0%
−32%
IGKV3−15 MM; n = 41
Fraction
100.0%
10.0%
2.3%
B
OAS
AL
MM
Probability
C
OAS conservation (Gini coefficient)
AL conservation (Gini coefficient)
MM conservation (Gini coefficient)
Correlation AL vs. OAS
Correlation AL vs. MM
D
IGKV3−15 AL: 13 MM: 41
Odds ratio (log scale)
Residue position
IGKV3−15 AL: 13 OAS: 449,473
Odds ratio (log scale)
Residue position
Enrichment
AL>OAS
None

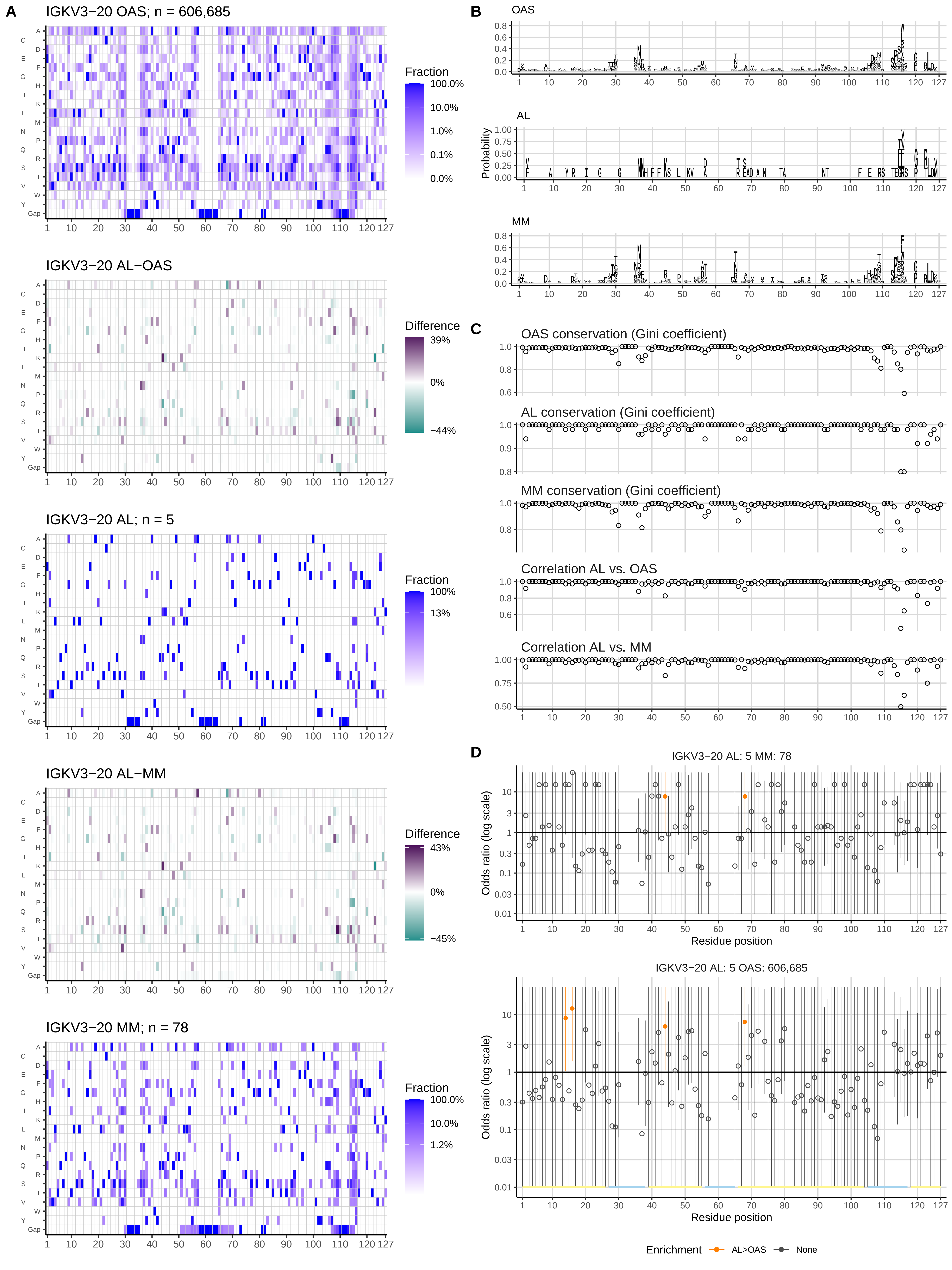
A
IGKV3−20 OAS; n = 606,685
Fraction
100.0%
10.0%
1.0%
0.1%
0.0%
IGKV3−20 AL−OAS
Difference
39%
0%
−44%
IGKV3−20 AL; n = 5
Fraction
100%
13%
IGKV3−20 AL−MM
Difference
43%
0%
−45%
IGKV3−20 MM; n = 78
Fraction
100.0%
10.0%
1.2%
A
C
D
E
F
G
H
I
K
L
M
N
P
Q
R
S
T
V
W
Y
Gap
B
OAS
AL
MM
Probability
C
OAS conservation (Gini coefficient)
AL conservation (Gini coefficient)
MM conservation (Gini coefficient)
Correlation AL vs. OAS
Correlation AL vs. MM
D
IGKV3−20 AL: 5 MM: 78
IGKV3−20 AL: 5 OAS: 606,685
Odds ratio (log scale)
Residue position
Enrichment
AL>OAS
None

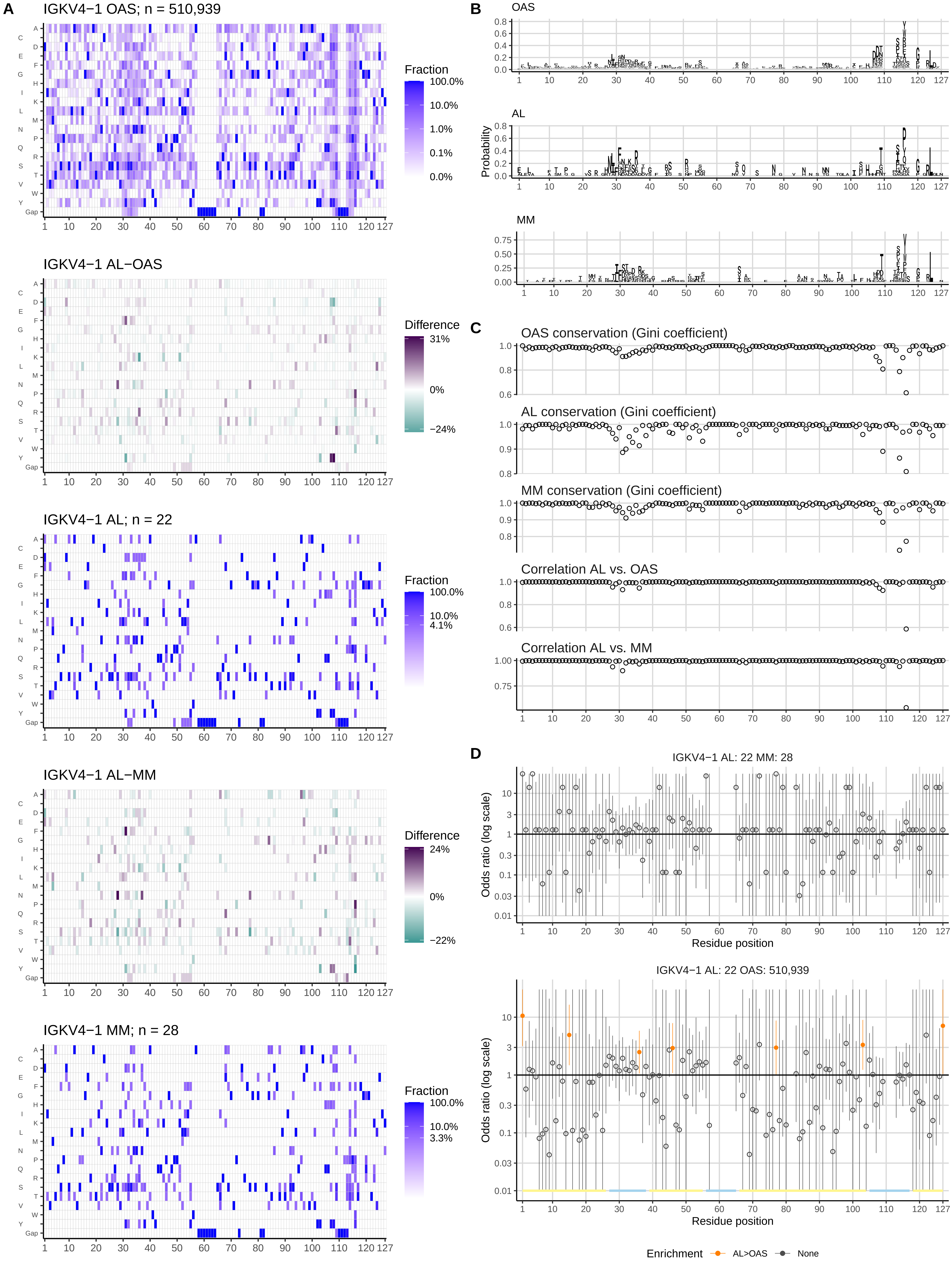
A
IGKV4−1 OAS; n = 510,939
Fraction
100.0%
10.0%
1.0%
0.1%
0.0%
IGKV4−1 AL−OAS
Difference
31%
0%
−24%
IGKV4−1 AL; n = 22
Fraction
100.0%
10.0%
4.1%
IGKV4−1 AL−MM
Difference
24%
0%
−22%
IGKV4−1 MM; n = 28
Fraction
100.0%
10.0%
3.3%
B
OAS
AL
MM
Probability
C
OAS conservation (Gini coefficient)
AL conservation (Gini coefficient)
MM conservation (Gini coefficient)
Correlation AL vs. OAS
Correlation AL vs. MM
D
IGKV4−1 AL: 22 MM: 28
Odds ratio (log scale)
Residue position
IGKV4−1 AL: 22 OAS: 510,939
Odds ratio (log scale)
Residue position
Enrichment
AL>OAS
None

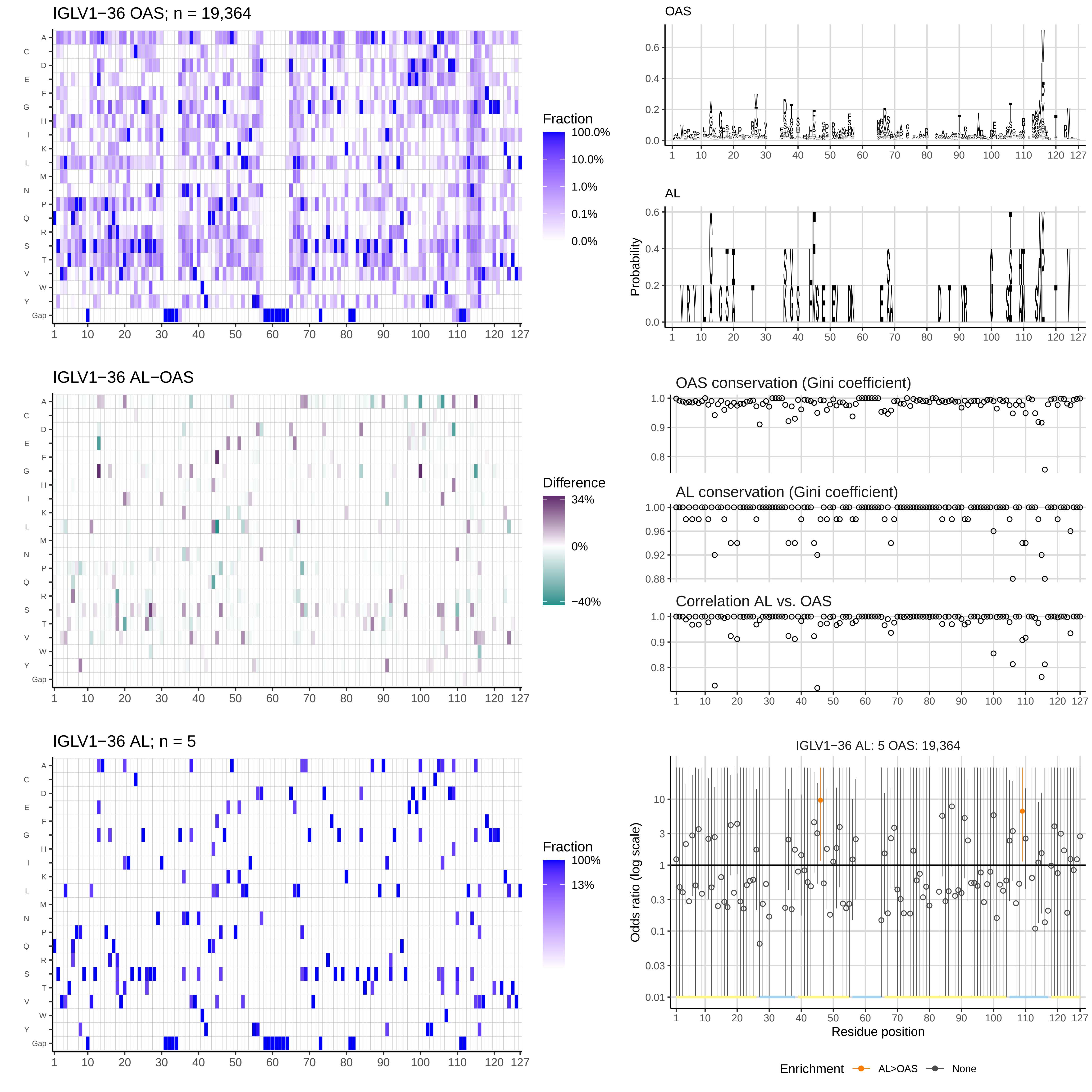

IGLV1−36 OAS; n = 19,364
Fraction
100.0%
10.0%
1.0%
0.1%
0.0%
OAS
AL
Probability
IGLV1−36 AL−OAS
Difference
34%
0%
−40%
OAS conservation (Gini coefficient)
AL conservation (Gini coefficient)
Correlation AL vs. OAS
IGLV1−36 AL; n = 5
Fraction
100%
13%
IGLV1−36 AL: 5 OAS: 19,364
Odds ratio (log scale)
Residue position
Enrichment
AL>OAS
None
A
C
D
E
F
G
H
I
K
L
M
N
P
Q
R
S
T
V
W
Y
Gap

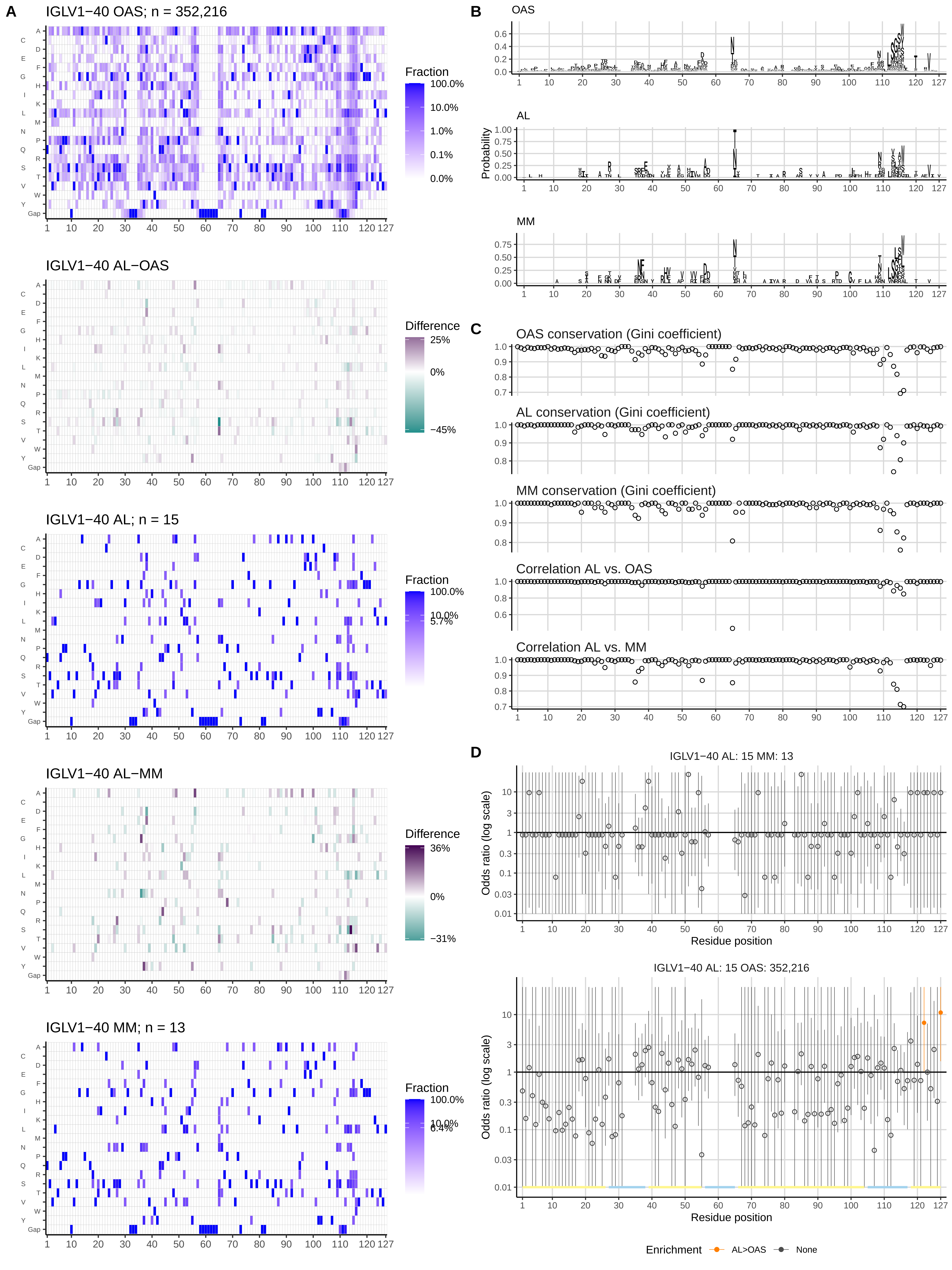

A
IGLV1−40 OAS; n = 352,216
Fraction
100.0%
10.0%
1.0%
0.1%
0.0%
IGLV1−40 AL−OAS
Difference
25%
0%
−45%
IGLV1−40 AL; n = 15
Fraction
100.0%
10.0%
5.7%
IGLV1−40 AL−MM
Difference
36%
0%
−31%
IGLV1−40 MM; n = 13
Fraction
100.0%
10.0%
6.4%
A C D E F G H I K L M N P Q R S T V W Y Gap
1 10 20 30 40 50 60 70 80 90 100 110 120 127
B
OAS
AL
MM
Probability
C
OAS conservation (Gini coefficient)
AL conservation (Gini coefficient)
MM conservation (Gini coefficient)
Correlation AL vs. OAS
Correlation AL vs. MM
D
IGLV1−40 AL: 15 MM: 13
Odds ratio (log scale)
Residue position
IGLV1−40 AL: 15 OAS: 352,216
Odds ratio (log scale)
Residue position
Enrichment
AL>OAS
None

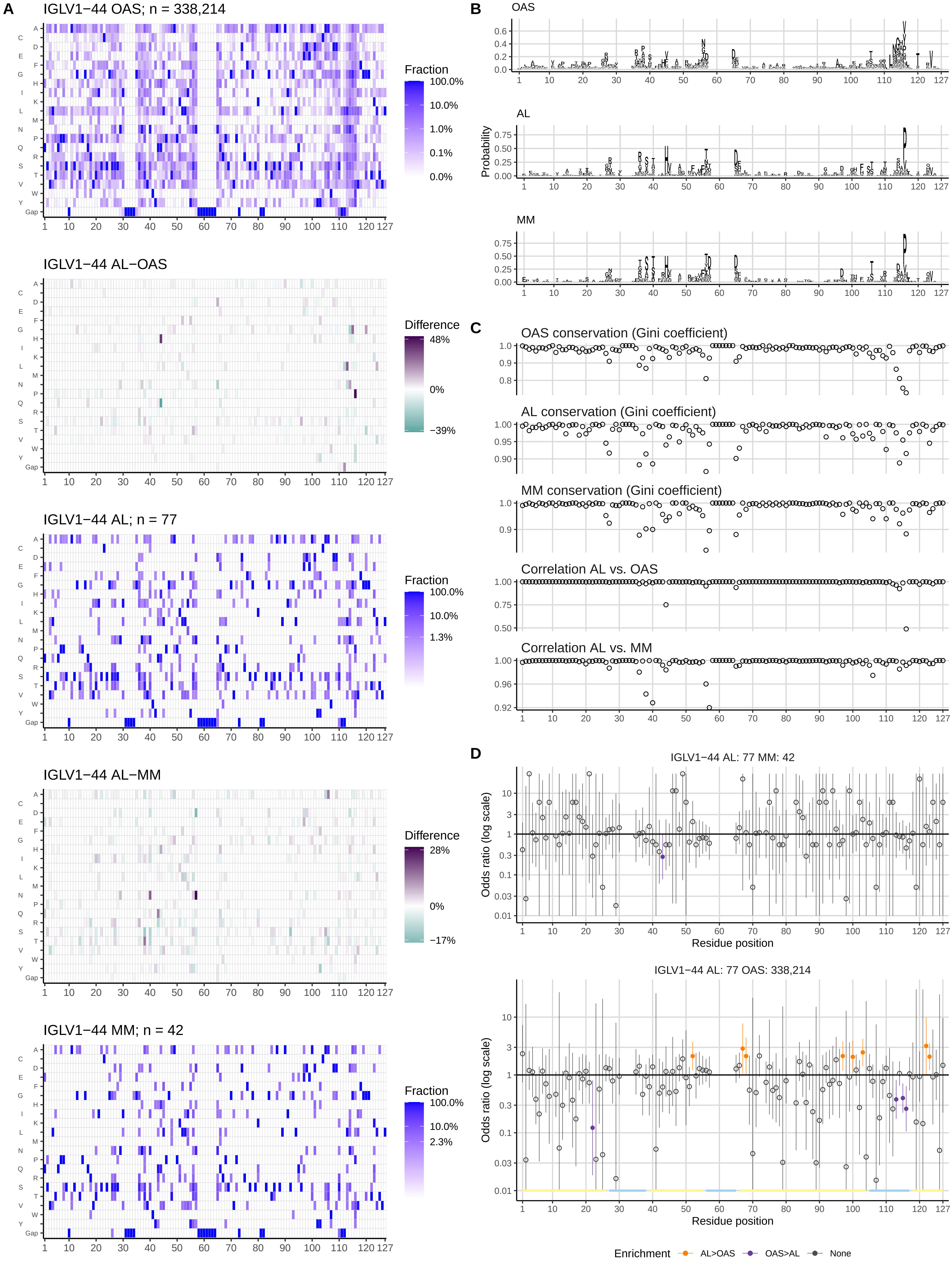
A
IGLV1−44 OAS; n = 338,214
Fraction
100.0%
10.0%
1.0%
0.1%
0.0%
IGLV1−44 AL−OAS
Difference
48%
0%
−39%
IGLV1−44 AL; n = 77
Fraction
100.0%
10.0%
1.3%
IGLV1−44 AL−MM
Difference
28%
0%
−17%
IGLV1−44 MM; n = 42
Fraction
100.0%
10.0%
2.3%
A C D E F G H I K L M N P Q R S T V W Y Gap
1 10 20 30 40 50 60 70 80 90 100 110 120 127
B
OAS
AL
MM
Probability
C
OAS conservation (Gini coefficient)
AL conservation (Gini coefficient)
MM conservation (Gini coefficient)
Correlation AL vs. OAS
Correlation AL vs. MM
D
IGLV1−44 AL: 77 MM: 42
Odds ratio (log scale)
Residue position
IGLV1−44 AL: 77 OAS: 338,214
Odds ratio (log scale)
Residue position
Enrichment
AL>OAS
OAS>AL
None

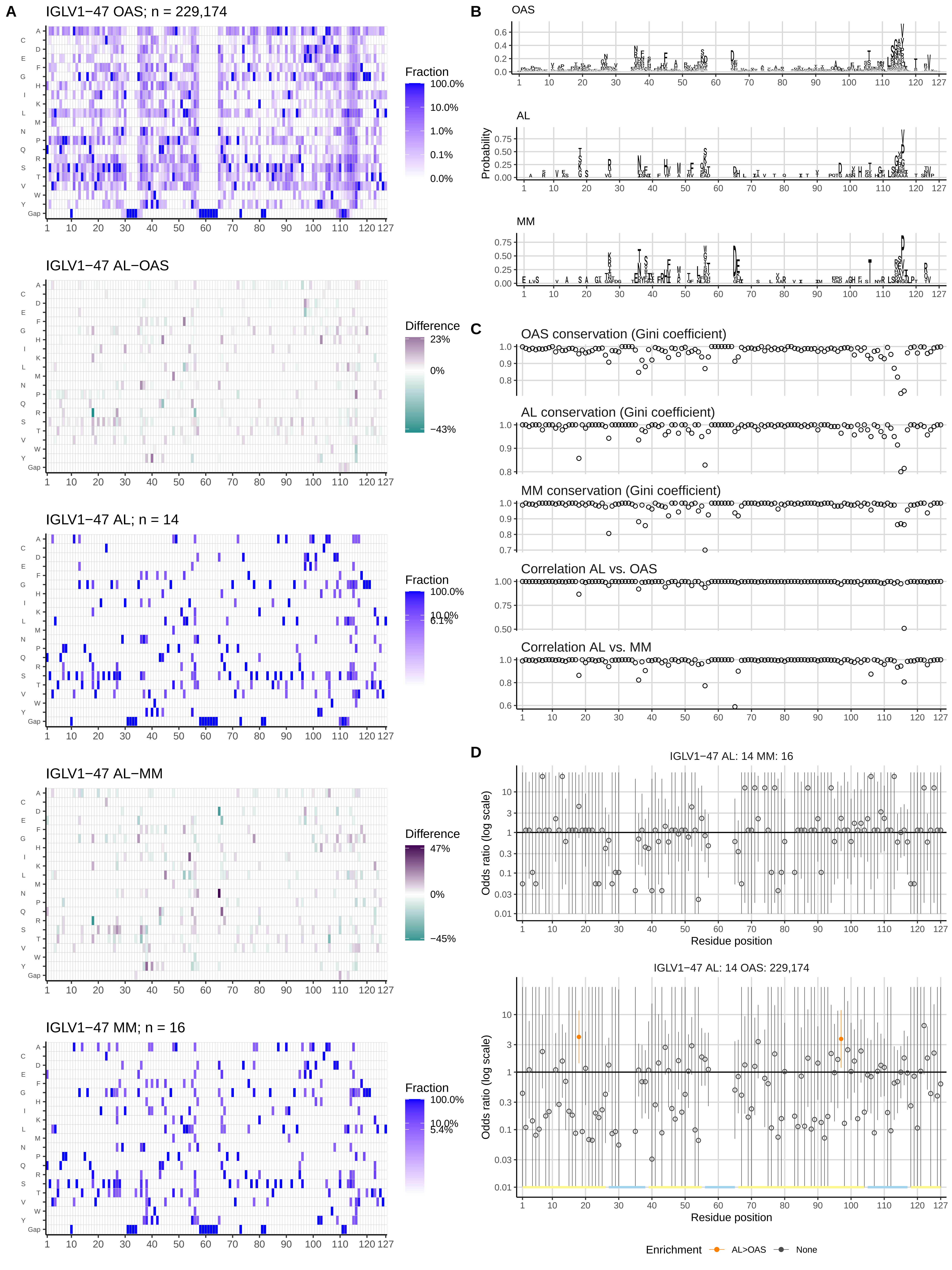
A
IGLV1−47 OAS; n = 229,174
Fraction
100.0%
10.0%
1.0%
0.1%
0.0%
IGLV1−47 AL−OAS
Difference
23%
0%
−43%
IGLV1−47 AL; n = 14
Fraction
100.0%
10.0%
6.1%
IGLV1−47 AL−MM
Difference
47%
0%
−45%
IGLV1−47 MM; n = 16
Fraction
100.0%
10.0%
5.4%
B
OAS
AL
MM
Probability
C
OAS conservation (Gini coefficient)
AL conservation (Gini coefficient)
MM conservation (Gini coefficient)
Correlation AL vs. OAS
Correlation AL vs. MM
D
IGLV1−47 AL: 14 MM: 16
Odds ratio (log scale)
Residue position
IGLV1−47 AL: 14 OAS: 229,174
Odds ratio (log scale)
Residue position
Enrichment
AL>OAS
None

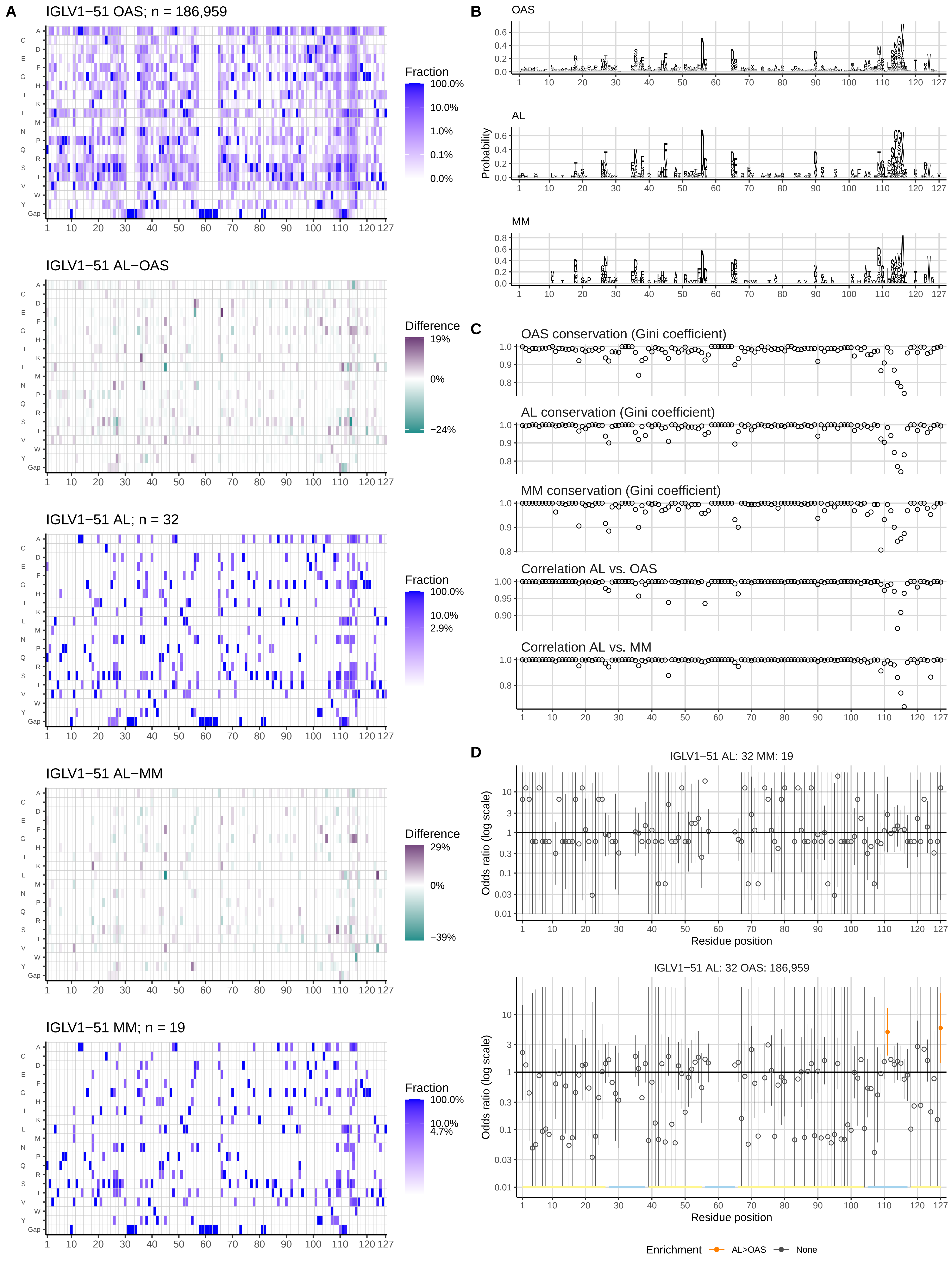
A
IGLV1−51 OAS; n = 186,959
Fraction
100.0%
10.0%
1.0%
0.1%
0.0%
IGLV1−51 AL−OAS
Difference
19%
0%
−24%
IGLV1−51 AL; n = 32
Fraction
100.0%
10.0%
2.9%
IGLV1−51 AL−MM
Difference
29%
0%
−39%
IGLV1−51 MM; n = 19
Fraction
100.0%
10.0%
4.7%
A
C
D
E
F
G
H
I
K
L
M
N
P
Q
R
S
T
V
W
Y
Gap
B
OAS
AL
MM
Probability
C
OAS conservation (Gini coefficient)
AL conservation (Gini coefficient)
MM conservation (Gini coefficient)
Correlation AL vs. OAS
Correlation AL vs. MM
D
IGLV1−51 AL: 32 MM: 19
Odds ratio (log scale)
Residue position
IGLV1−51 AL: 32 OAS: 186,959
Odds ratio (log scale)
Residue position
Enrichment
AL>OAS
None

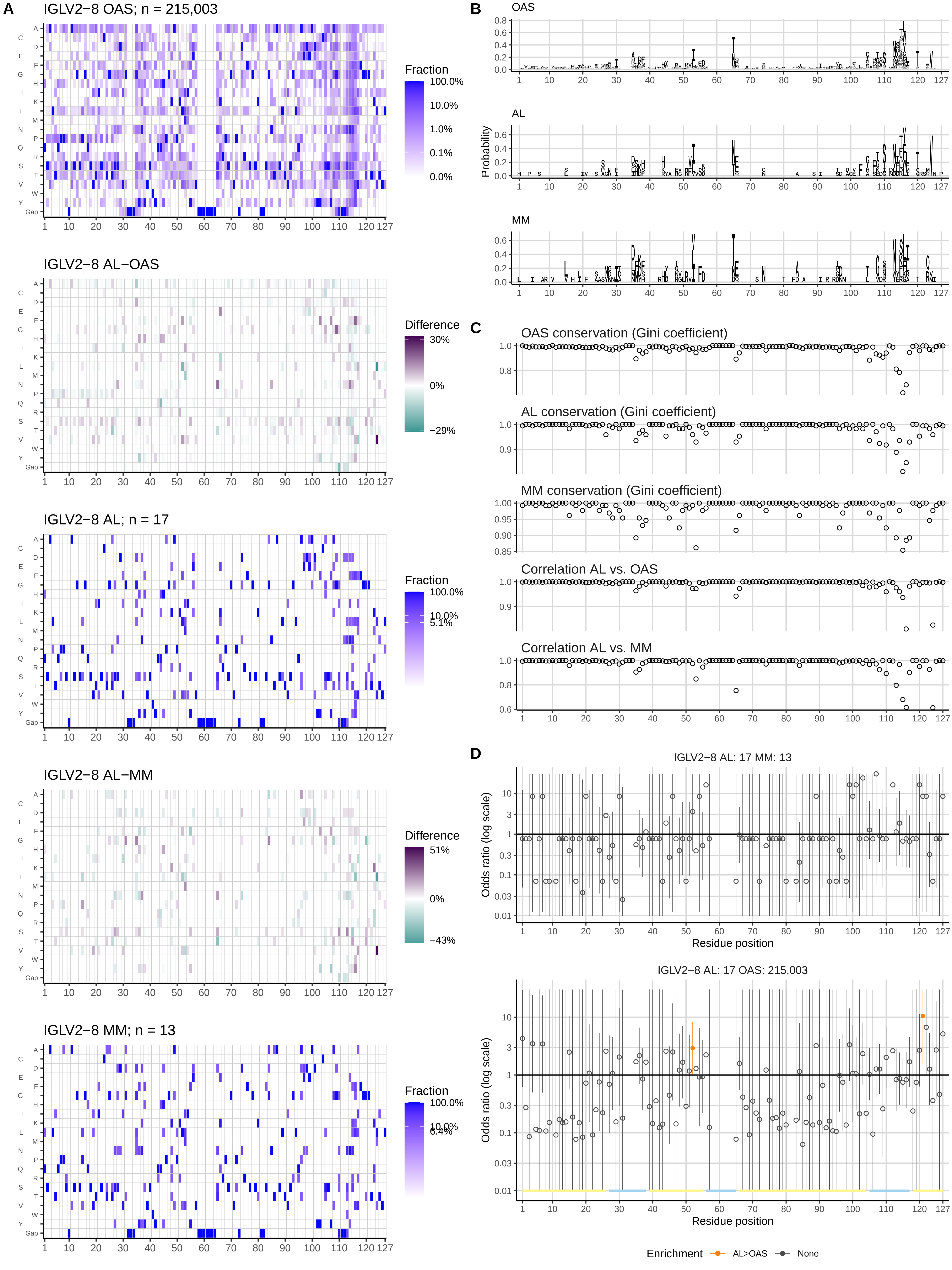

A
IGLV2−8 OAS; n = 215,003
IGLV2−8 AL−OAS
IGLV2−8 AL; n = 17
IGLV2−8 AL−MM
IGLV2−8 MM; n = 13
Fraction
Difference
B
OAS
AL
MM
Probability
C
OAS conservation (Gini coefficient)
AL conservation (Gini coefficient)
MM conservation (Gini coefficient)
Correlation AL vs. OAS
Correlation AL vs. MM
D
IGLV2−8 AL: 17 MM: 13
IGLV2−8 AL: 17 OAS: 215,003
Odds ratio (log scale)
Residue position
Enrichment
AL>OAS
None

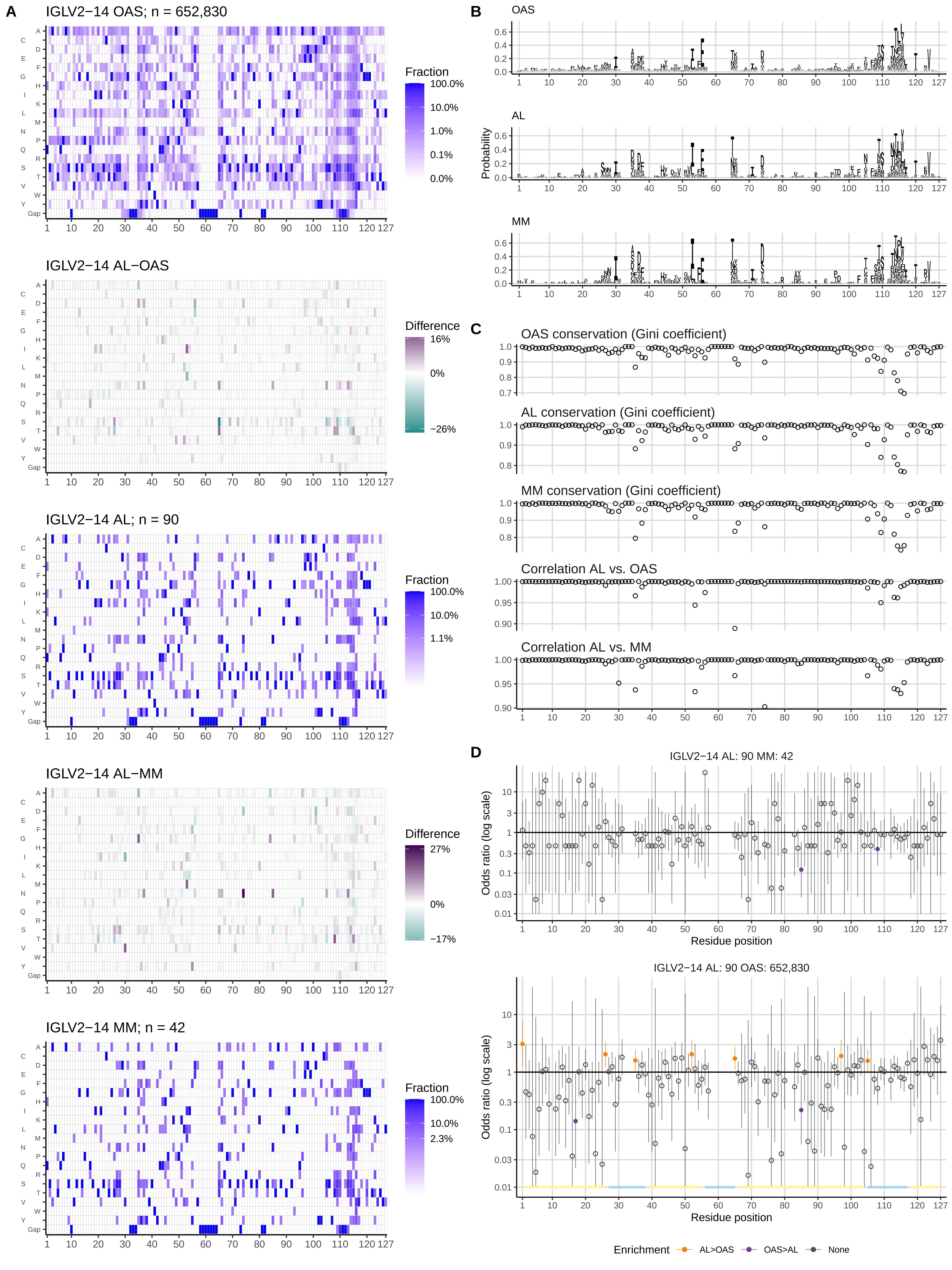

A
IGLV2−14 OAS; n = 652,830
Fraction
100.0%
10.0%
1.0%
0.1%
0.0%
IGLV2−14 AL−OAS
Difference
16%
0%
−26%
IGLV2−14 AL; n = 90
Fraction
100.0%
10.0%
1.1%
IGLV2−14 AL−MM
Difference
27%
0%
−17%
IGLV2−14 MM; n = 42
Fraction
100.0%
10.0%
2.3%
B
OAS
AL
MM
Probability
C
OAS conservation (Gini coefficient)
AL conservation (Gini coefficient)
MM conservation (Gini coefficient)
Correlation AL vs. OAS
Correlation AL vs. MM
D
IGLV2−14 AL: 90 MM: 42
Odds ratio (log scale)
Residue position
IGLV2−14 AL: 90 OAS: 652,830
Odds ratio (log scale)
Residue position
Enrichment
AL>OAS
OAS>AL
None

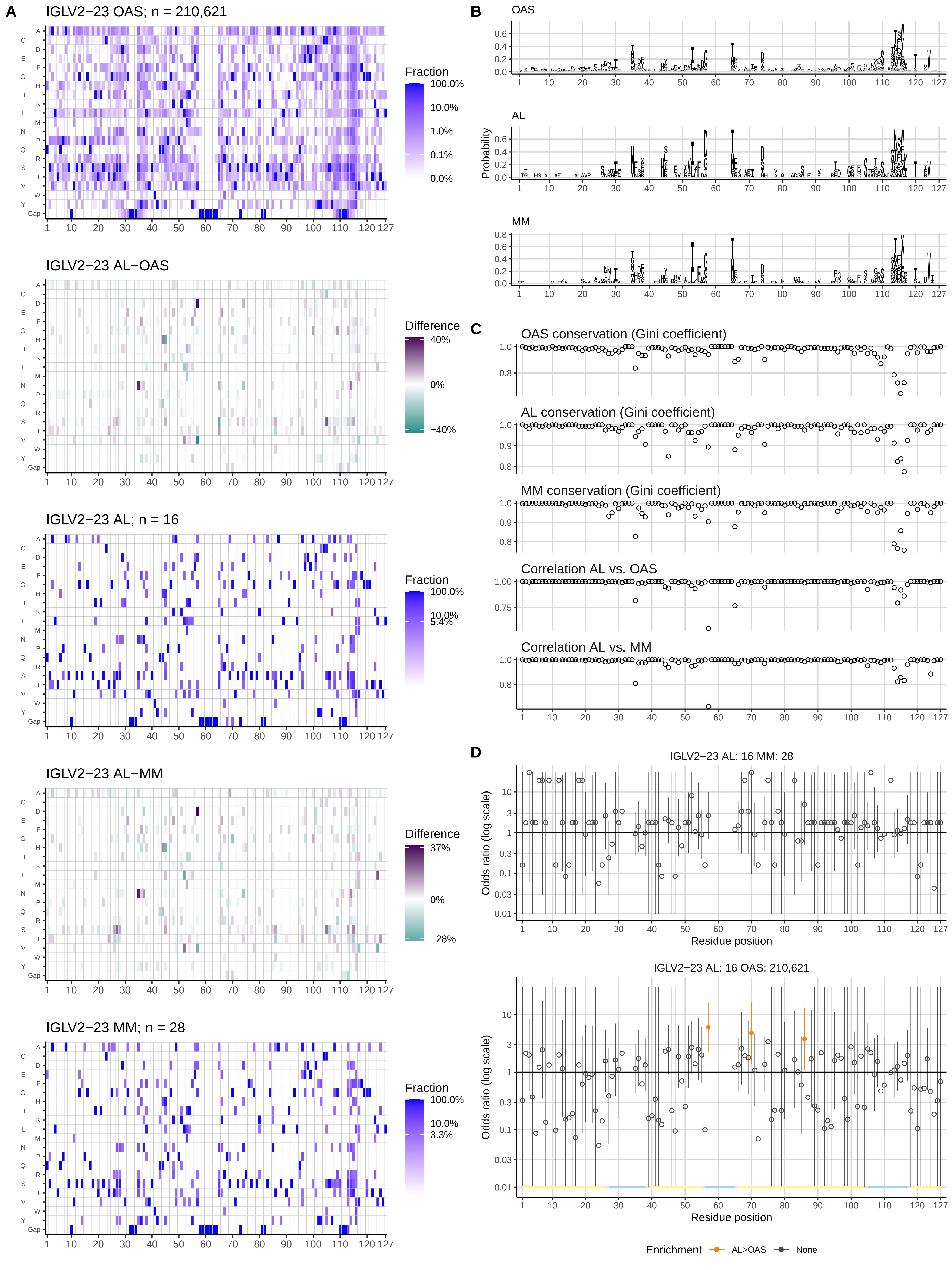
A
IGLV2−23 OAS; n = 210,621
Fraction
100.0%
10.0%
1.0%
0.1%
0.0%
IGLV2−23 AL−OAS
Difference
40%
0%
−40%
IGLV2−23 AL; n = 16
Fraction
100.0%
10.0%
5.4%
IGLV2−23 AL−MM
Difference
37%
0%
−28%
IGLV2−23 MM; n = 28
Fraction
100.0%
10.0%
3.3%
A C D E F G H I K L M N P Q R S T V W Y Gap
1 10 20 30 40 50 60 70 80 90 100 110 120 127
B
OAS
AL
MM
Probability
C
OAS conservation (Gini coefficient)
AL conservation (Gini coefficient)
MM conservation (Gini coefficient)
Correlation AL vs. OAS
Correlation AL vs. MM
D
IGLV2−23 AL: 16 MM: 28
IGLV2−23 AL: 16 OAS: 210,621
Odds ratio (log scale)
Residue position
Enrichment
AL>OAS
None

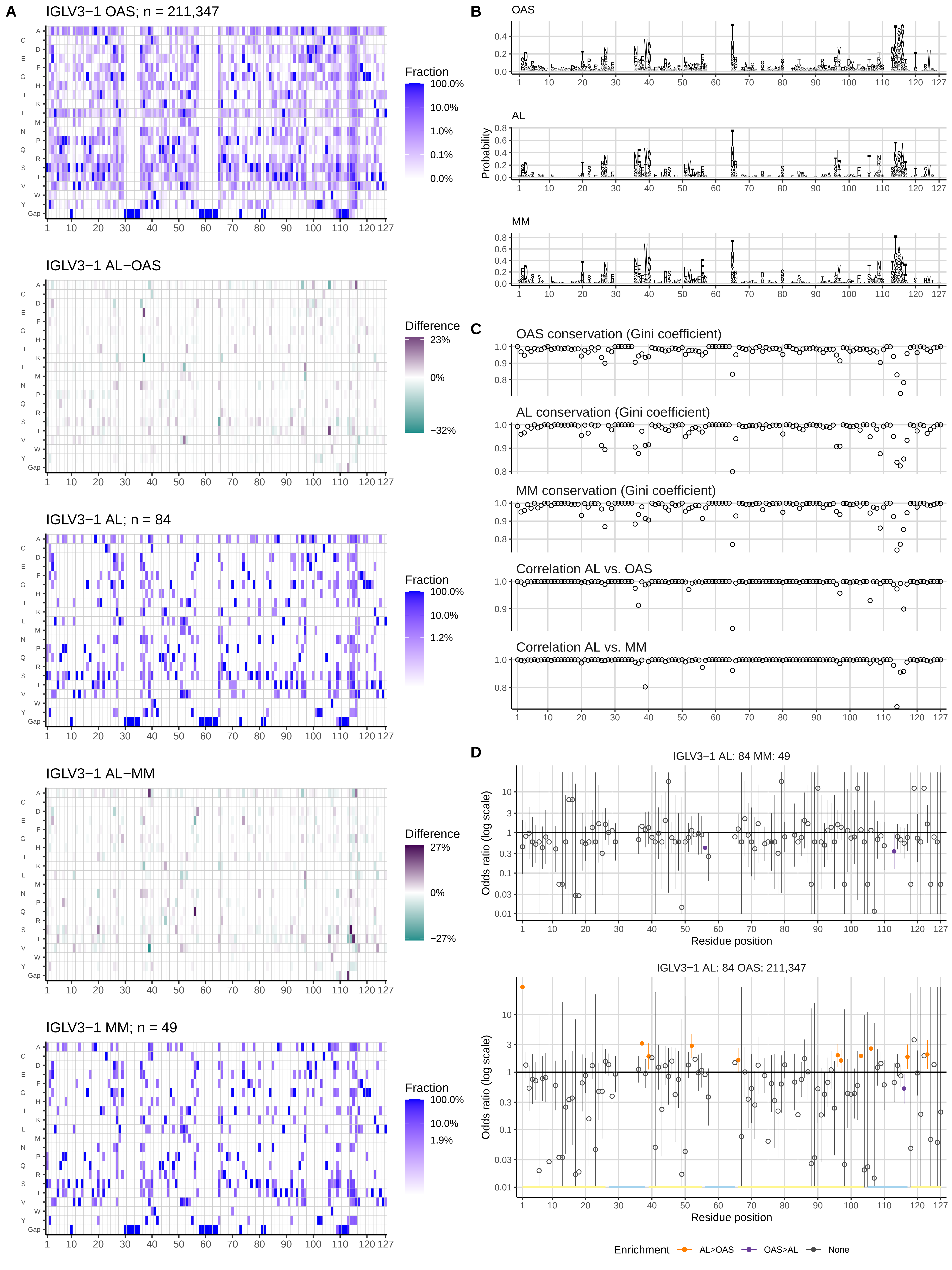
A
IGLV3−1 OAS; n = 211,347
Fraction
100.0%
10.0%
1.0%
0.1%
0.0%
IGLV3−1 AL−OAS
Difference
23%
0%
−32%
IGLV3−1 AL; n = 84
Fraction
100.0%
10.0%
1.2%
IGLV3−1 AL−MM
Difference
27%
0%
−27%
IGLV3−1 MM; n = 49
Fraction
100.0%
10.0%
1.9%
C D E F G H I K L M N P Q R S T V W Y Gap
1 10 20 30 40 50 60 70 80 90 100 110 120 127
B
OAS
AL
MM
Probability
C
OAS conservation (Gini coefficient)
AL conservation (Gini coefficient)
MM conservation (Gini coefficient)
Correlation AL vs. OAS
Correlation AL vs. MM
D
IGLV3−1 AL: 84 MM: 49
IGLV3−1 AL: 84 OAS: 211,347
Odds ratio (log scale)
Residue position
Enrichment
AL>OAS
OAS>AL
None

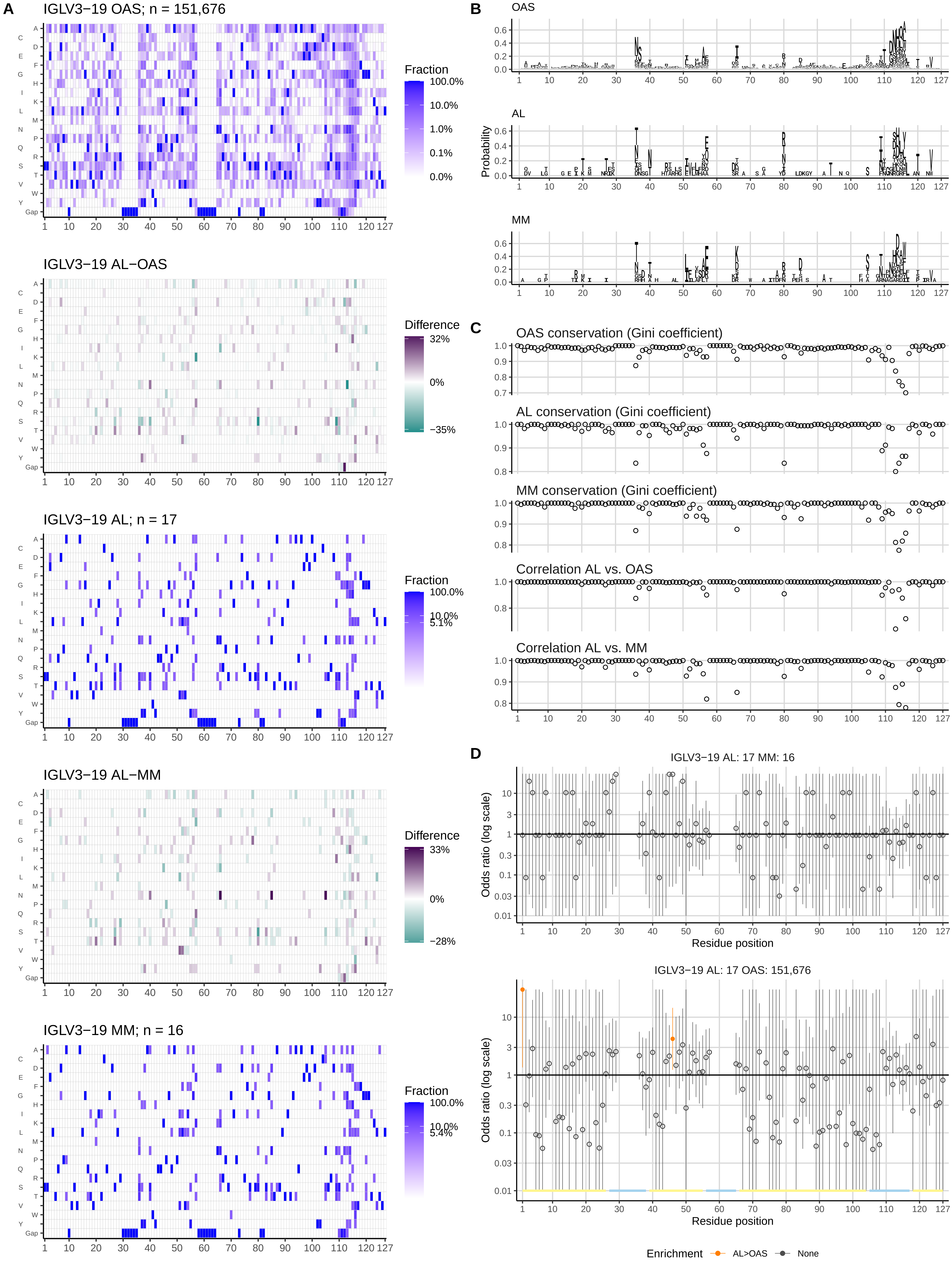
A
IGLV3−19 OAS; n = 151,676
Fraction
100.0%
10.0%
1.0%
0.1%
0.0%
IGLV3−19 AL−OAS
Difference
32%
0%
−35%
IGLV3−19 AL; n = 17
Fraction
100.0%
10.0%
5.1%
IGLV3−19 AL−MM
Difference
33%
0%
−28%
IGLV3−19 MM; n = 16
Fraction
100.0%
10.0%
5.4%
A C D E F G H I K L M N P Q R S T V W Y Gap
B
OAS
AL
MM
Probability
C
OAS conservation (Gini coefficient)
AL conservation (Gini coefficient)
MM conservation (Gini coefficient)
Correlation AL vs. OAS
Correlation AL vs. MM
D
IGLV3−19 AL: 17 MM: 16
Odds ratio (log scale)
Residue position
IGLV3−19 AL: 17 OAS: 151,676
Odds ratio (log scale)
Residue position
Enrichment
AL>OAS
None

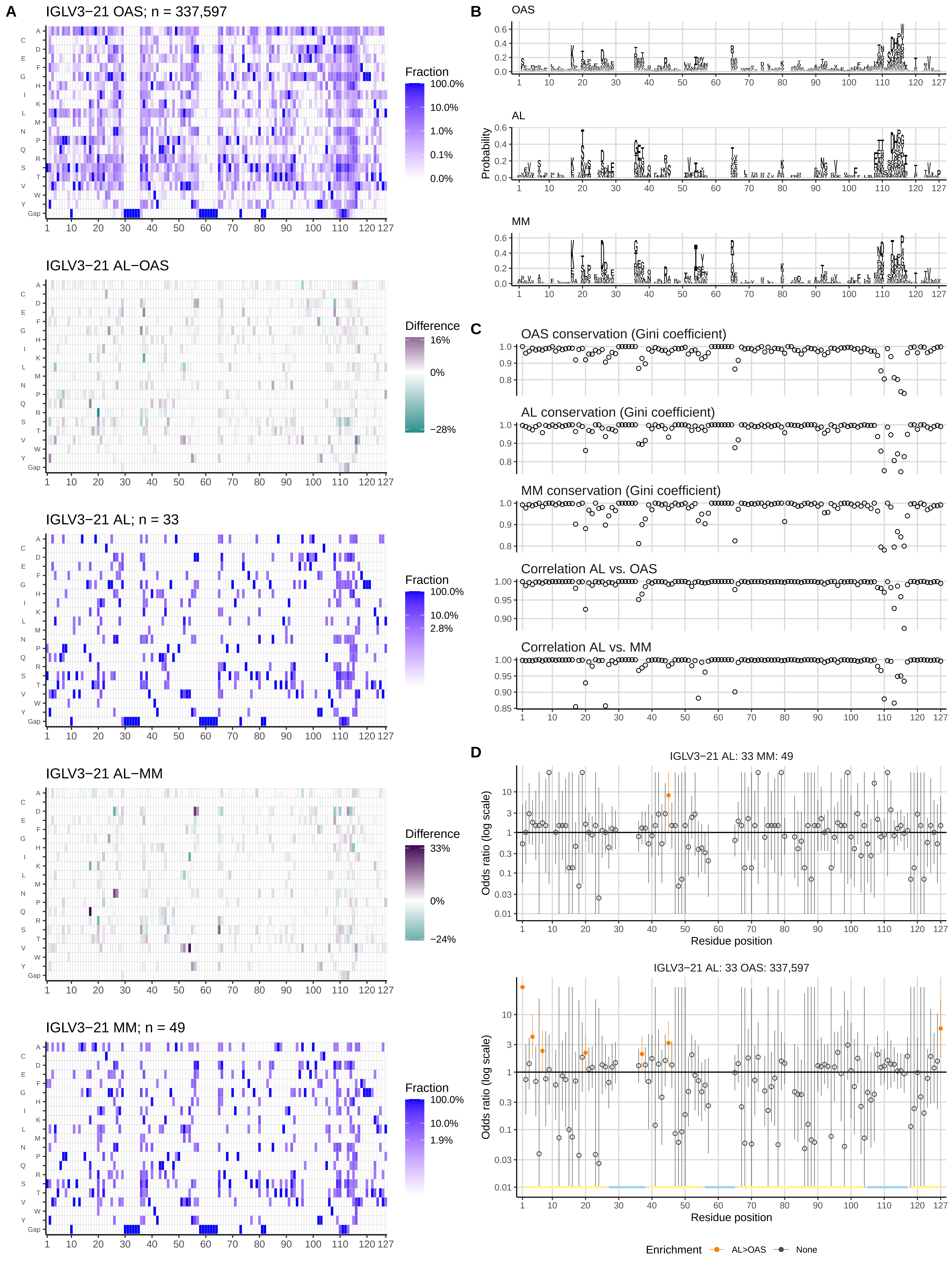
A
IGLV3−21 OAS; n = 337,597
Fraction
100.0%
10.0%
1.0%
0.1%
0.0%
IGLV3−21 AL−OAS
Difference
16%
0%
−28%
IGLV3−21 AL; n = 33
Fraction
100.0%
10.0%
2.8%
IGLV3−21 AL−MM
Difference
33%
0%
−24%
IGLV3−21 MM; n = 49
Fraction
100.0%
10.0%
1.9%
Gap
B
OAS
AL
MM
Probability
C
OAS conservation (Gini coefficient)
AL conservation (Gini coefficient)
MM conservation (Gini coefficient)
Correlation AL vs. OAS
Correlation AL vs. MM
D
IGLV3−21 AL: 33 MM: 49
Odds ratio (log scale)
Residue position
IGLV3−21 AL: 33 OAS: 337,597
Odds ratio (log scale)
Residue position
Enrichment
AL>OAS
None

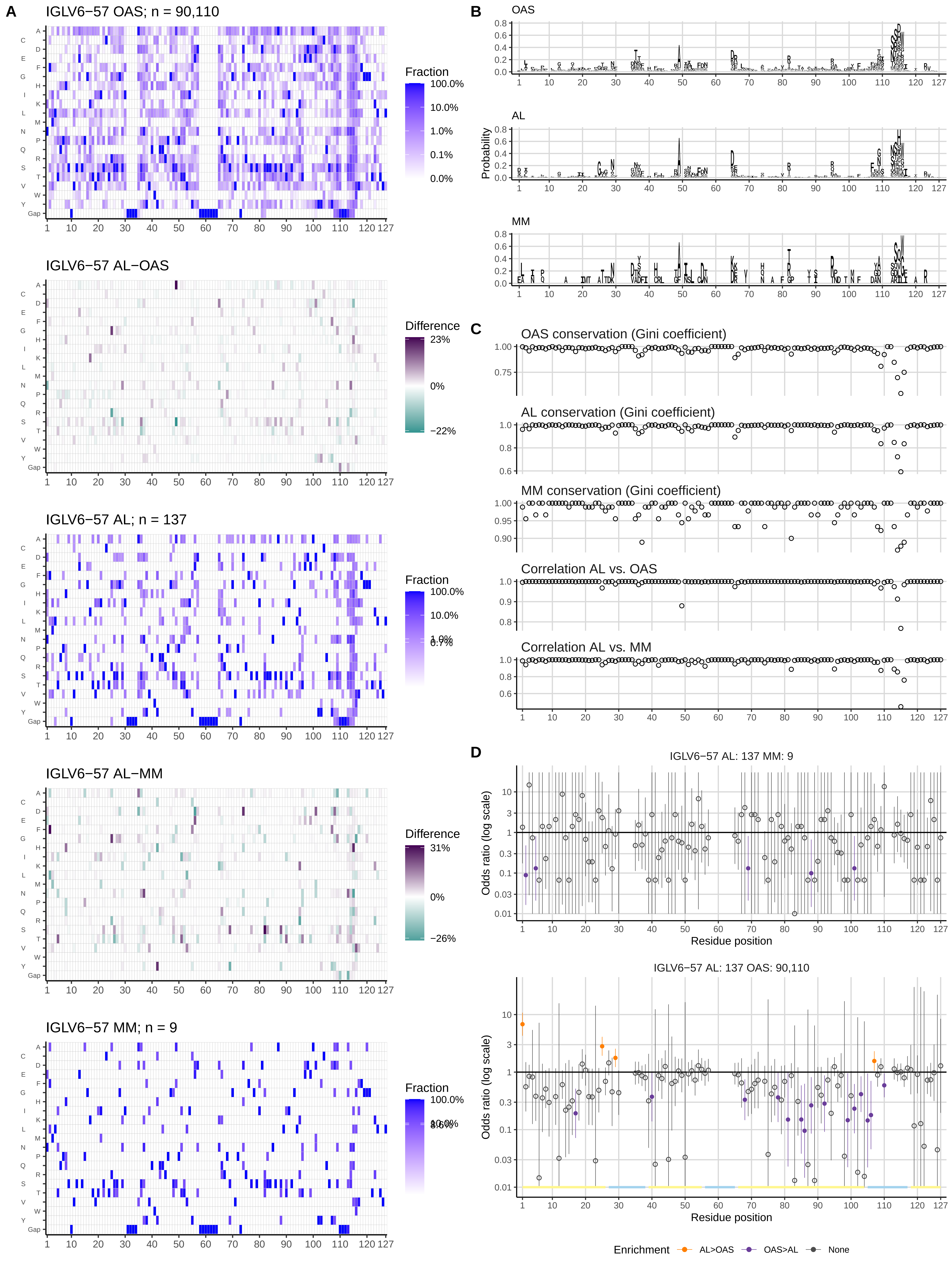
A
IGLV6−57 OAS; n = 90,110
IGLV6−57 AL−OAS
IGLV6−57 AL; n = 137
IGLV6−57 AL−MM
IGLV6−57 MM; n = 9
Fraction
Difference
B
OAS
AL
MM
Probability
C
OAS conservation (Gini coefficient)
AL conservation (Gini coefficient)
MM conservation (Gini coefficient)
Correlation AL vs. OAS
Correlation AL vs. MM
D
IGLV6−57 AL: 137 MM: 9
IGLV6−57 AL: 137 OAS: 90,110
Odds ratio (log scale)
Residue position
Enrichment
AL>OAS
OAS>AL
None
