# Supplementary material for: Clone-specific residue changes at multiple positions are associated with amyloid formation by antibody light chains
Source: Front Immunol. 2025 Aug 1;16:1622207. doi: 10.3389/fimmu.2025.1622207 (PMC12375997; doi:10.3389/fimmu.2025.1622207)
Supplement: Supplementary file 2 [file SupplementaryFile1.pdf]

Supplementary Material

Clone-specific residue changes at multiple positions are associated with amyloid formation by antibody light chains

Gareth J. Morgan*, Tatiana Prokaeva

*** Correspondence:** Gareth J. Morgan: gjmorgan@bu.edu

1 Supplementary Data

Supplementary Data Sheet 1 (PDF): Summary of data for each of the 20 IGV_L genes studied. Each figure contains the same panels. A) Consensus matrix and difference matrix heatmaps, as in Figure 1. B) Sequence logo plots showing the probability of observing residues at each position within each group of LC sequences. The most frequent residue observed at each position in the OAS consensus matrices is excluded from the plots. C) Residue conservation and correlation between sets of LCs at each position in the consensus matrices. Conservation measured by the Gini coefficient, where 0 is equal proportions of all residues, including gaps, and 1 is absolute conservation. Correlations are measured by the Pearson correlation coefficients (ρ) between fractions of residues at each position for the AL *vs.* MM and AL *vs.* OAS comparisons. A value of 1 corresponds to identical fractions of every residue, including gaps. D) Odds ratios for frequencies of uncommon residues, as in Figure 4.

Supplementary Table 1 (Excel): OAS consensus matrices for each of the 20 IGV_L genes studied. The worksheets show the relative frequency of each residue at all IMGT positions for the OAS sequences assigned to the IGV_L gene. All alleles and IGJ_L genes were considered together, so the matrices incorporate the distributions of alleles and IGJ_L genes within the original OAS data.

Supplementary Table 2 (Excel): Positions enriched in uncommon mutations for the AL *vs.* MM and AL *vs.* OAS comparisons. Structural data for each residue is derived from the germline V_L-domain models shown in Supplementary Figure 3. Secondary structure and solvent accessible surface area (SASA) were assigned using DSSP. Counts of sequences harboring uncommon or common residues at each position are shown for each set of LCs.

Supplementary Table 3 (Excel): Extended data for the previously identified residue positions shown in Tables 1-3. Structural data for each residue is derived from the germline V_L-domain models shown in Supplementary Figure 3. Secondary structure and solvent accessible surface area (SASA) were assigned using DSSP. Counts of sequences harboring uncommon or common residues at each position are shown for each set of LCs.

2 Supplementary Figures 1-7

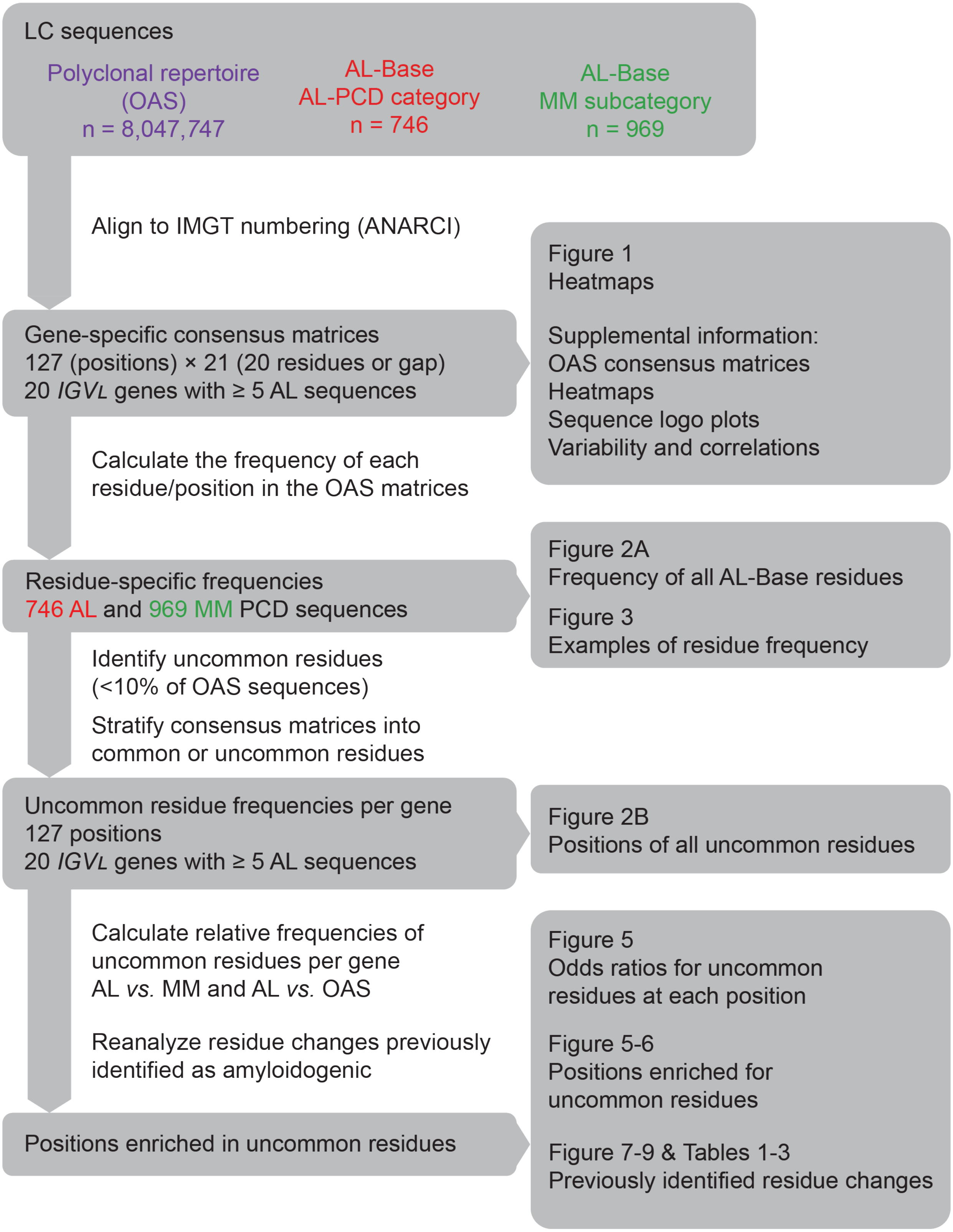

Supplementary Figure 1: Overview of analysis workflow.

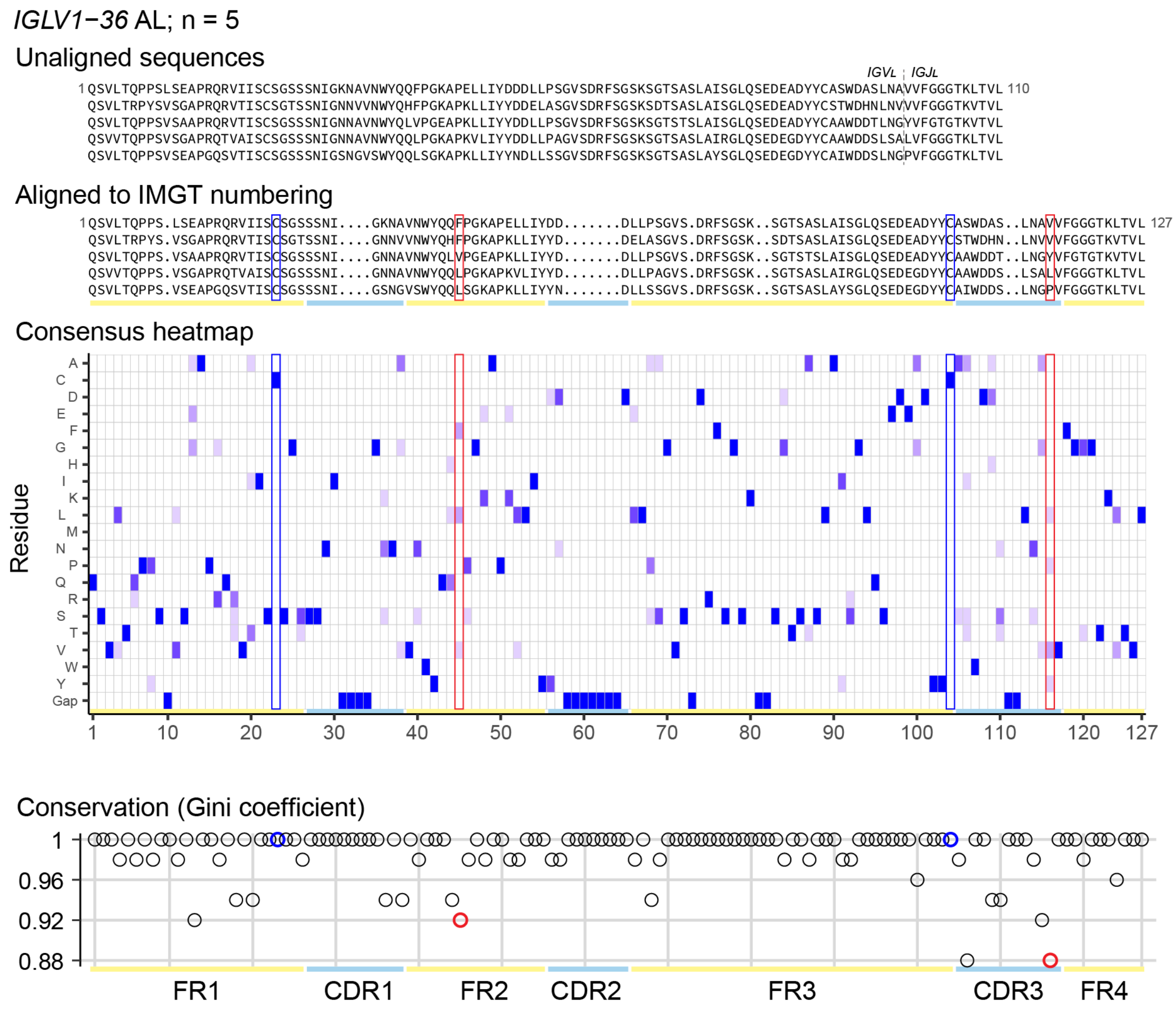

Supplementary Figure 2: Example of consensus matrices using the five available AL LCs derived from *IGLV1-36*. Alignment to IMGT numbering allows calculation of a consensus matrix without the requirement to calculate pairwise distances between sequences. The consensus matrix is shown as a heatmap, as in Figure 1. The Gini coefficient, shown in the bottom panel, is calculated at each residue position of the matrix. To illustrate the difference between conserved and variable residues, examples are highlighted with boxes in the consensus matrix and colored symbols in the Gini coefficient plot. Two conserved cysteine residues that form an internal disulfide bond are highlighted in blue, and two variable residues in FR2 and CDR3 are highlighted in red.

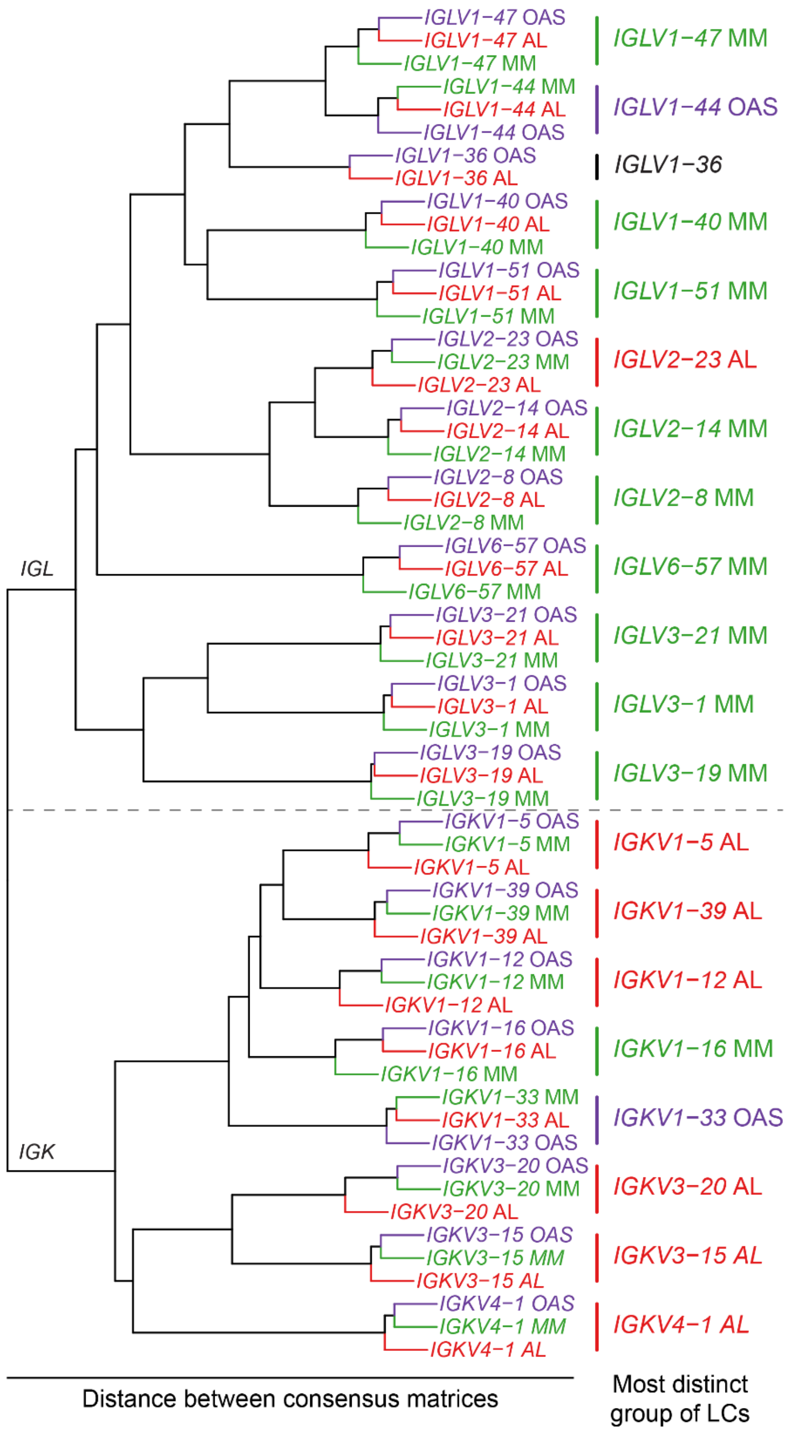

Supplementary Figure 3: Dendrogram showing the sequence similarity between groups of LCs, calculated from the pairwise distances between the consensus matrices exemplified in Figure 1. The length of the line connecting each node is proportional to the Euclidian distance between the corresponding matrices. The outlying group is highlighted at the right in all cases, except for *IGLV1-36*, for which no MM LCs are deposited in AL-Base. Within each gene, one of the three sets of LCs is (inevitably) an outlier, with a greater distance to the other two sets. AL LCs were the most distinct group in 6 of the 8 *IGKV* genes studied. In contrast, among 11 *IGLV* genes (excluding *IGLV1-36*, for which no MM LCs are present in the AL-Base data), MM LCs are the most distinct group in 9 cases.

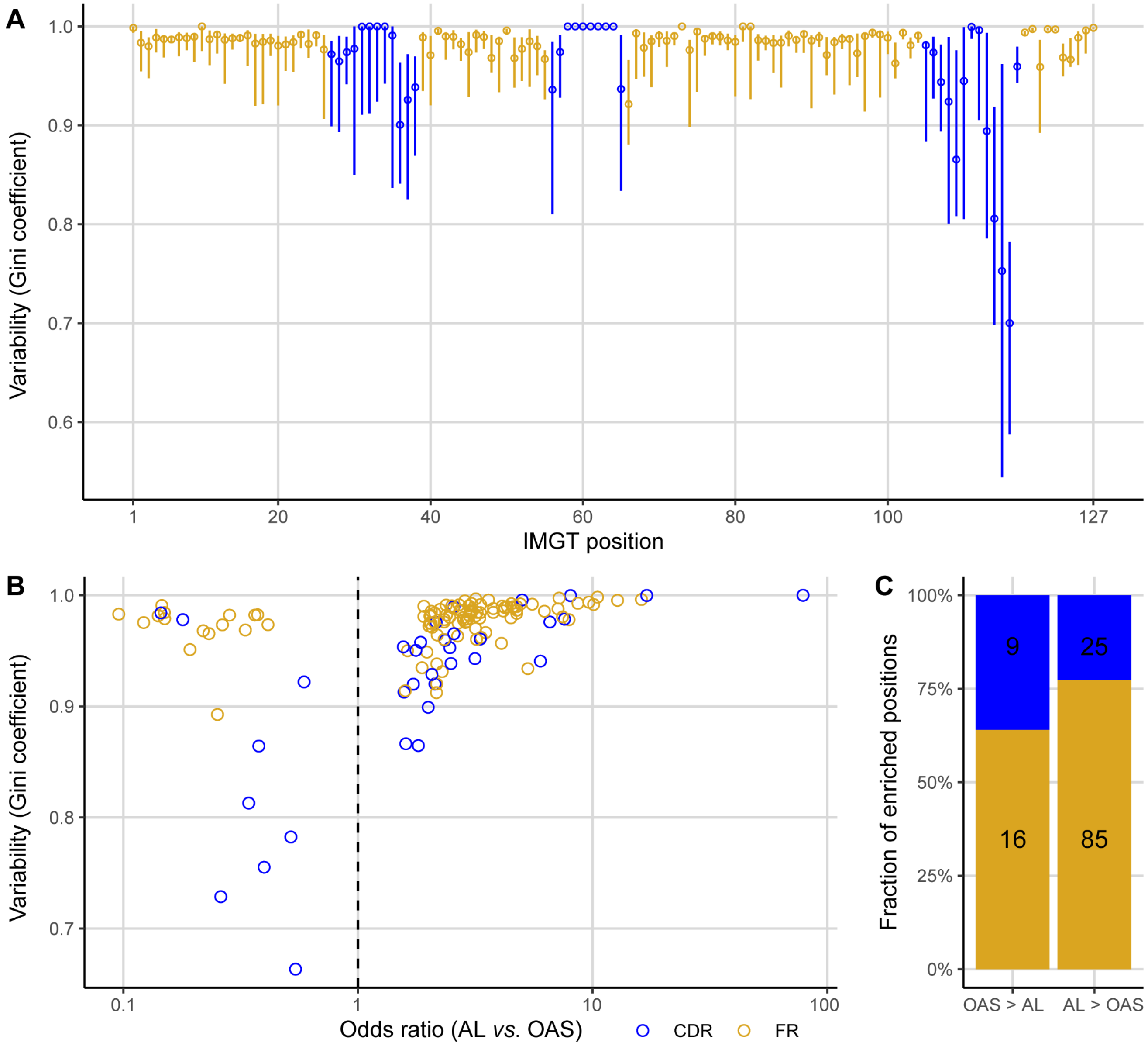

Supplementary Figure 4: Positions enriched in uncommon residues (see Figure 5 in the main text) are relatively conserved in the OAS repertoire. FR positions are shown in yellow and CDR positions shown in blue. A) Variable conservation across OAS LC sequences. For each of the 20 IGV_L genes analyzed, the Gini coefficient was calculated at each position (Supplementary Data 1). The median and range of these Gini coefficients are shown as points and lines, respectively. A Gini coefficient of 1 represents absolute conservation of a single residue at each position. CDR residues are less conserved than FR residues, but there is variation within and between IGV_L genes at all occupied LC positions. Note that positions 58-64 are defined by IMGT as conserved gaps in LC sequences. B) Correlation between enrichment in uncommon residues and conservation among OAS LCs. We identified 135 positions among 20 IGV_L genes where uncommon residues were significantly more or less frequent in AL *vs.* OAS LCs, excluding 6 V_L domain N-termini and 9 V_L domain C-termini ($p < 0.05$, Figure 5). The odds ratio for observing an uncommon residue in the AL *vs.* OAS comparison is plotted against the Gini coefficient for that position in the gene-specific OAS

consensus matrix. Uncommon residues were most frequently enriched at positions that were conserved in OAS LCs. C) Distribution between FRs and CDRs of positions enriched in uncommon residues in the AL *vs.* OAS comparison (n = 135). Most positions where uncommon residues are less (left bar) or more (right bar) frequent in AL LCs occur within FRs.

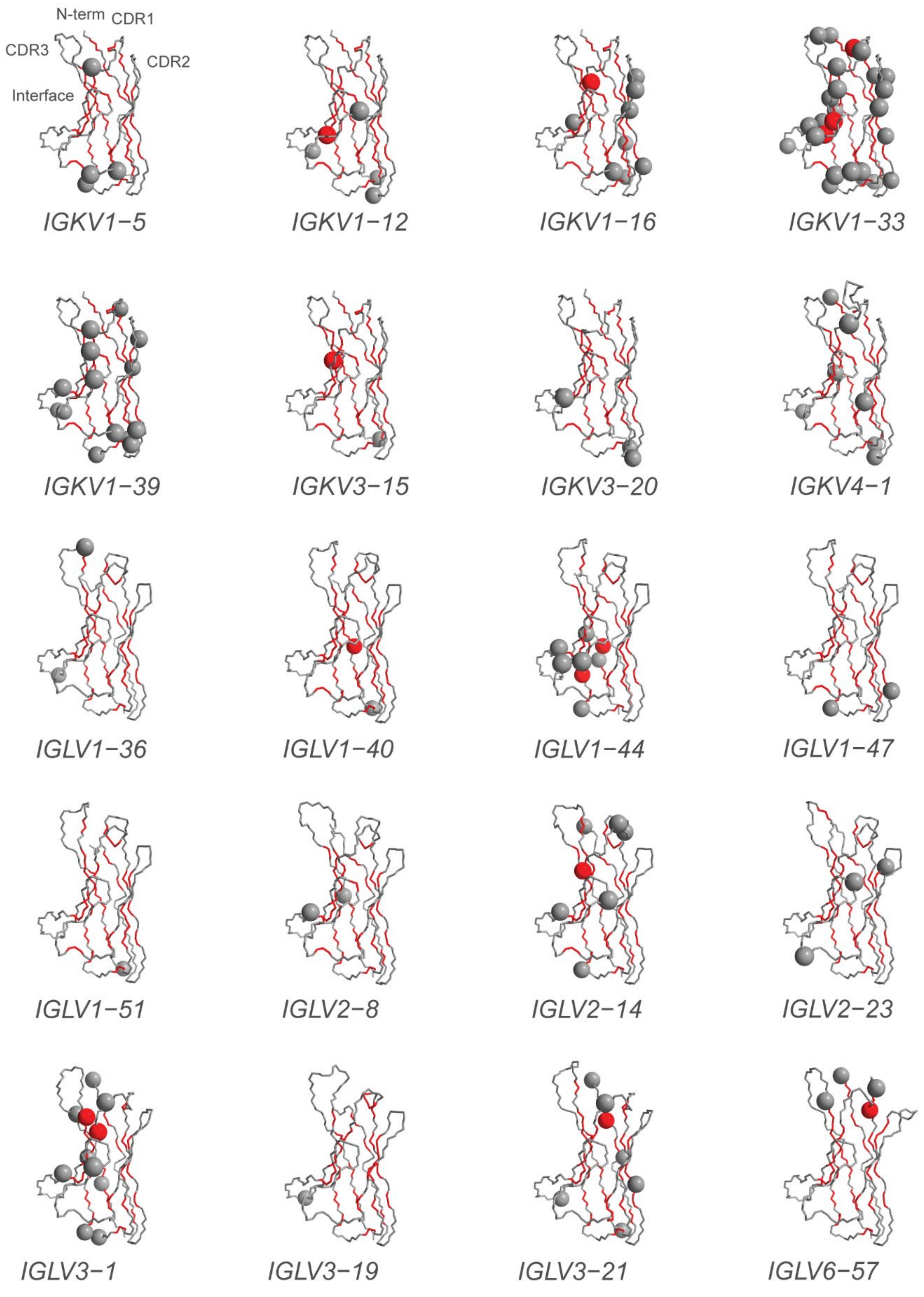

Supplementary Figure 5: Positions where uncommon residues are more frequent in AL LCs *vs.* OAS LCs (orange panels in Figure 7) are shown as spheres on *in silico* models of the corresponding V_L-domain. Solvent exposed residues are colored grey and buried residues are colored red.

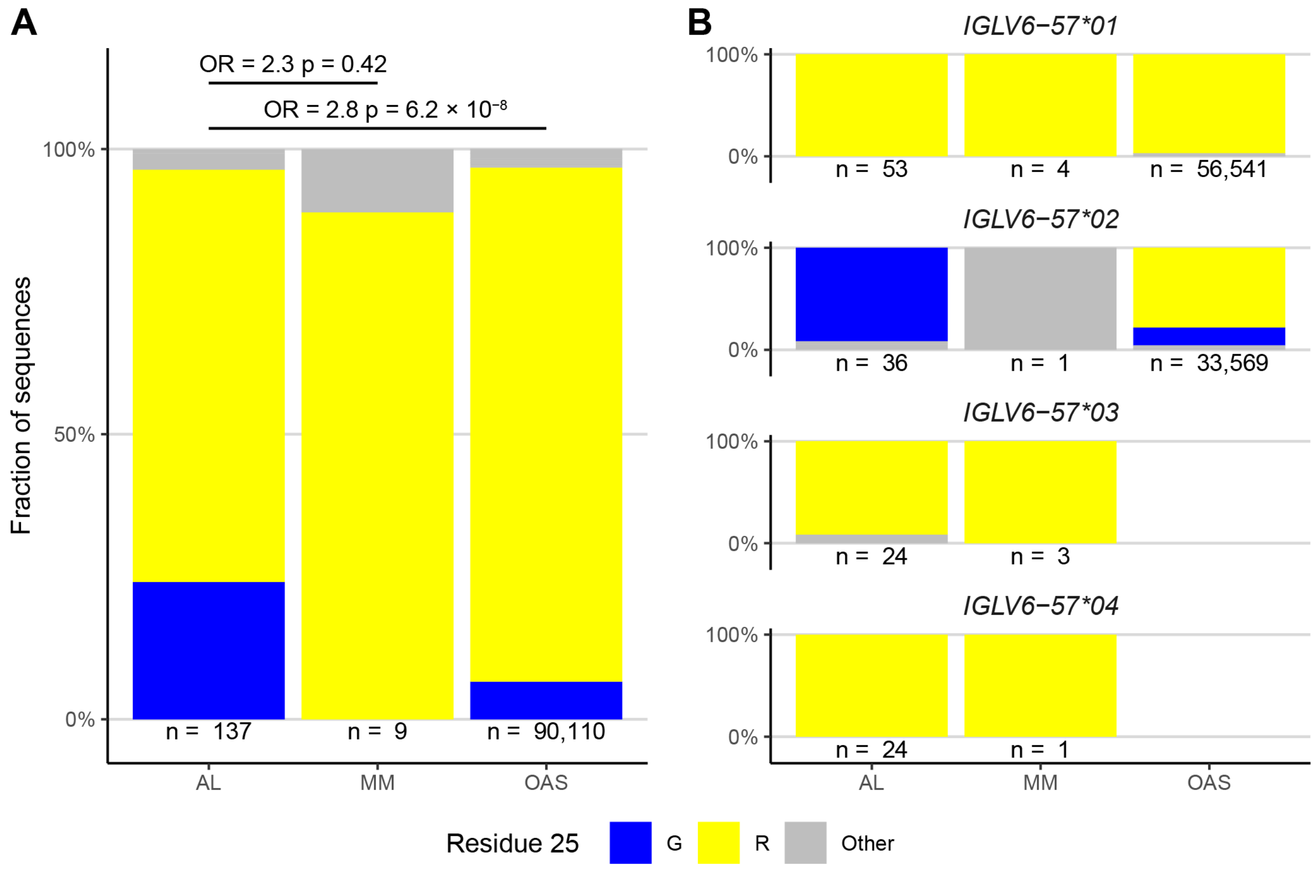

Supplementary Figure 6: The *IGLV6-57* R25G polymorphism is associated with AL amyloidosis. Bars show the fraction of *IGLV6-57*-derived LCs harboring an argenine (R), glycine (G) or other residue at position 25. A) All *IGLV6-57*-derived LCs. B) LCs stratified by their assigned allele.

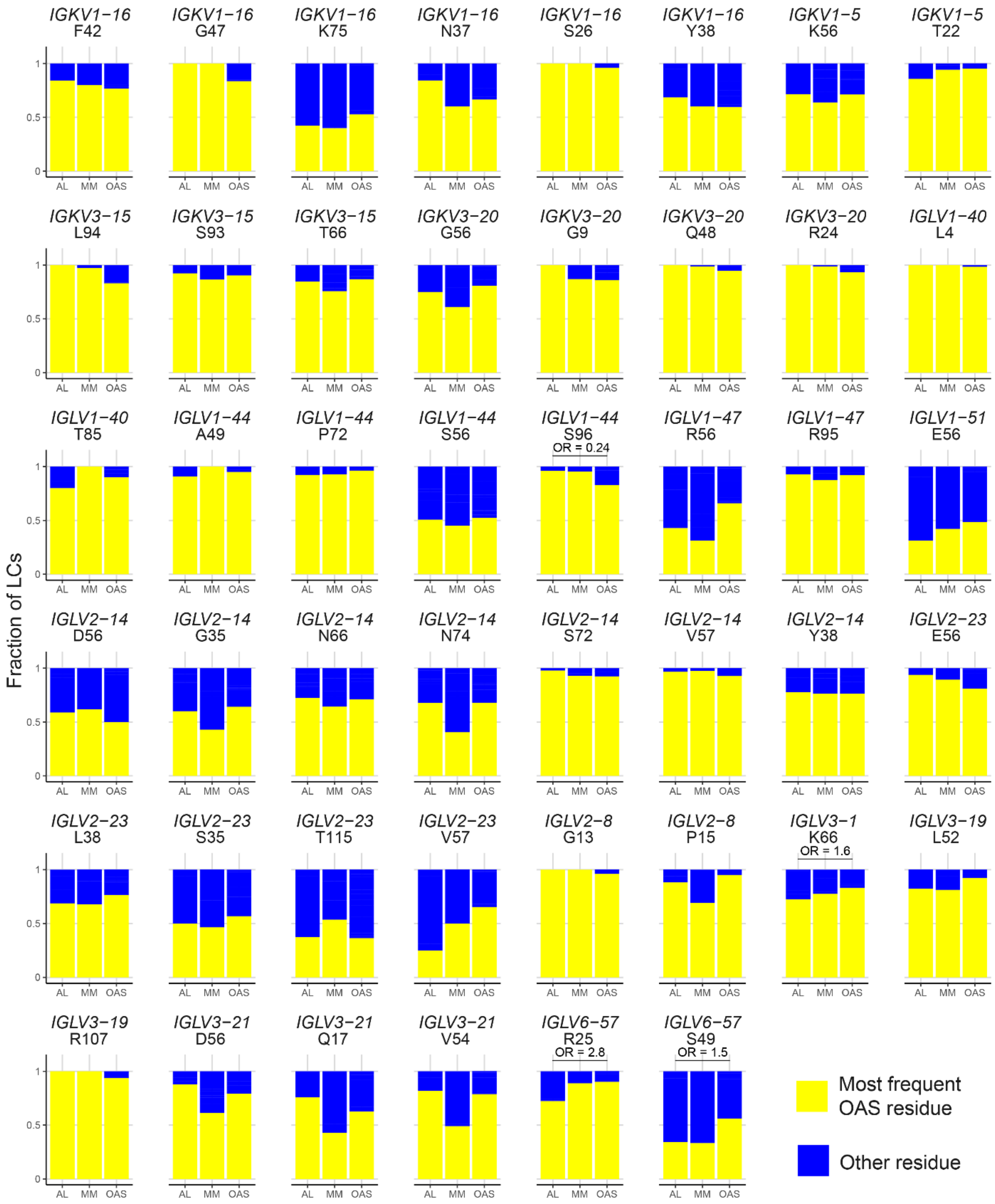

Supplementary Figure 7: Fractions of residues at sites that vary between alleles of *IGV*$_L$ genes. The most frequent residue in the OAS consensus matrices is shown for each position. Odds ratios (OR) for AL *vs.* OAS comparisons that reach significance ($p < 0.05$) without correction for multiple testing are shown. Only the OR for *IGLV6-57* R25 remains significant after correction for multiple testing (FDR = 2.84×10^{-6}).
